# Supplementary material for: Synthesis of Fluorescent, Dumbbell-Shaped Polyurethane Homo- and Heterodendrimers and Their Photophysical Properties
Source: Int J Mol Sci. 2023 Jan 14;24(2):1662. doi: 10.3390/ijms24021662 (PMC9866862; doi:10.3390/ijms24021662)
Supplement: Supplementary file 1 [file ijms-24-01662-s001.zip › ijms-2108617-supplementary.pdf]

SUPPORTING INFORMATION

Synthesis of Fluorescent, Dumbbell-Shaped Polyurethane Homo- and Heterodendrimers and Their Photophysical Properties

Dhruba P. Poudel and Richard T. Taylor *

Department of Chemistry and Biochemistry, Miami University, Oxford, OH 45056, USA
* Correspondence: taylorrt@miamioh.edu

Table of Contents

S**1. Experimental section..S3**

a. ***Equations for the synthesis of G1 dendron 3 using one-pot multicomponent Curtius reaction*** *.................S3*

b. ***Equation for azide-alkyne click reaction forming blue - fluorescent G1 dendron 6*** *...................................S3*

c. ***Equations for the synthesis of 4-azido-N-ethyl-1,8-naphthalimide 5*** *...S4*

d. ***Equation for the synthesis of naphthalimide clicked G1 dendron 7*** *...S5*

e. ***Equation for the synthesis of a mixture of G1 homo- and heterodendrimers*** *..S5*

f. ***Equations showing growth of dendrons 12-16*** *...S6*

g. ***Equation showing late-stage modification of polyurethane dendrons 14 and 16*** *.....................................S7*

h. ***Equation for the synthesis of G2 dendrimers 24-26*** *...S8*

i. ***Equation for the synthesis of G3 dendrimers and their expanded structures*** *..S9*

S2. Spectral data of polyurethane dendrons and dendrimers S11

S1 Experimental section

a. Equations for the synthesis of G1 dendron 3 using one-pot multicomponent Curtius reaction

Scheme S1. Synthesis of dendron 3.

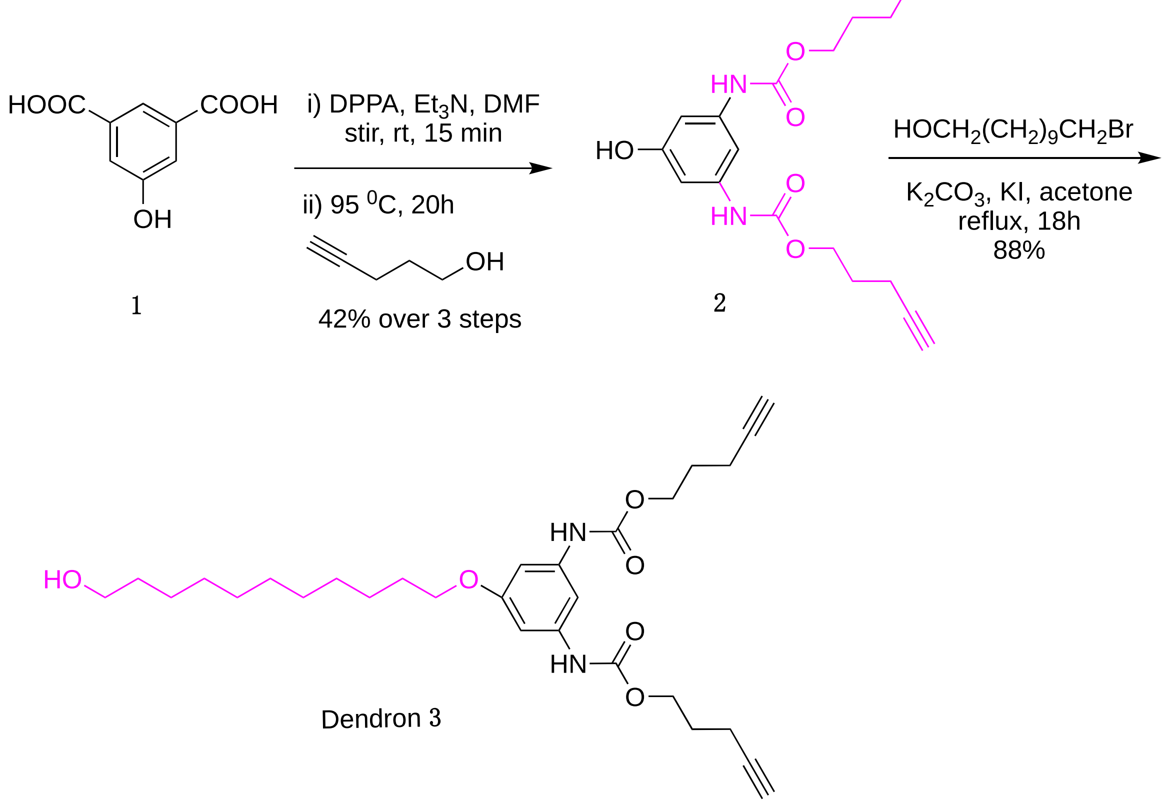

b. Equation for azide-alkyne click reaction forming blue - fluorescent G1 dendron 6

Scheme S2. Synthesis of blue fluorescent dendron 6.

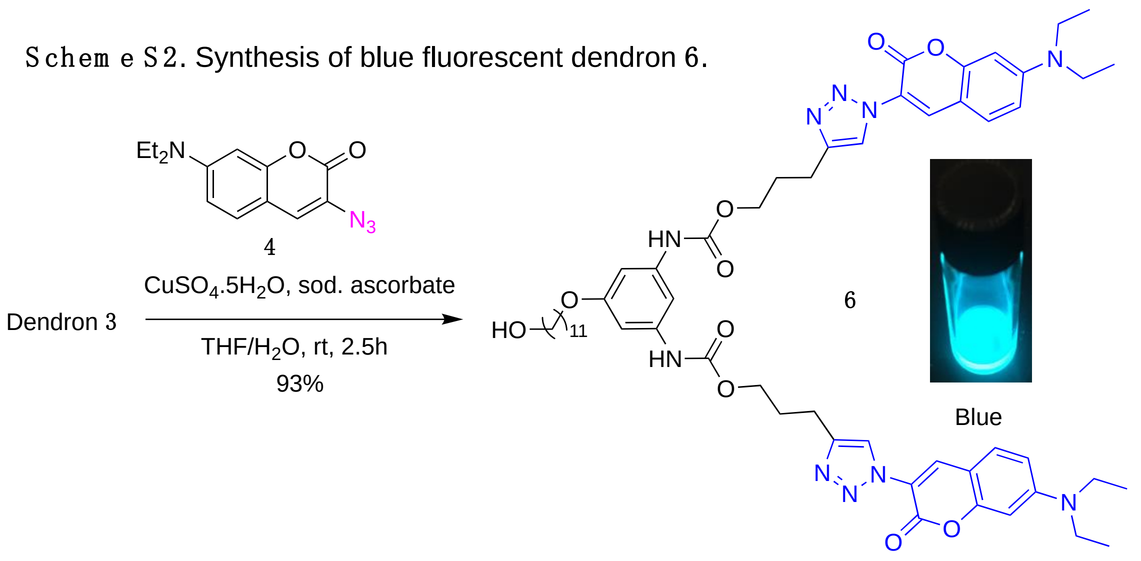

c. Equations for the synthesis of 4-azido-N-ethyl-1,8-naphthalimide 5

Scheme S3. Synthesis of 4-azido-N-ethyl-1,8-naphthalimide, 17

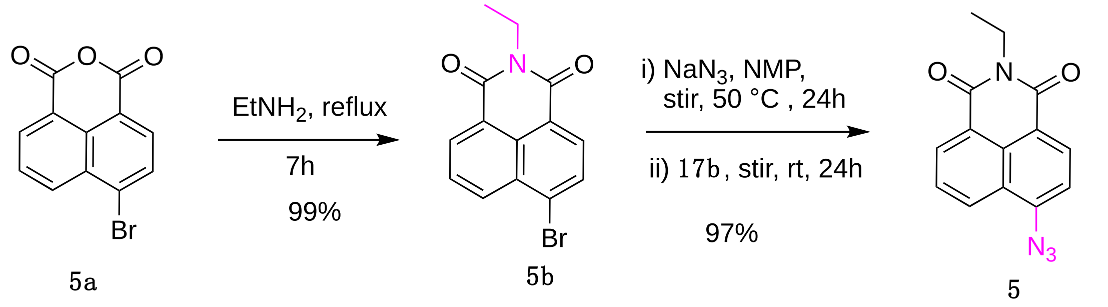

d. Equation for the synthesis of naphthalimide clicked G1 dendron 7

Scheme S4. Synthesis of G1 dendron 7.

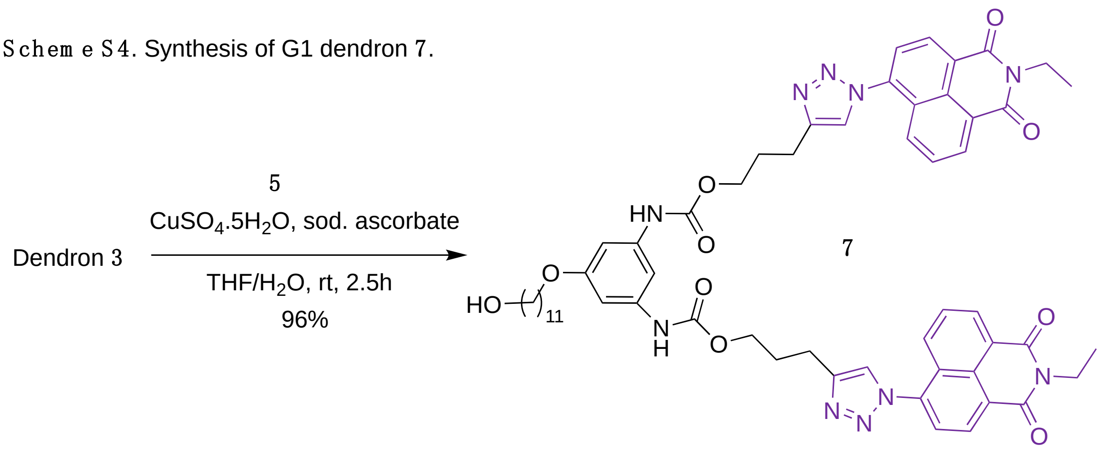

e. Equation for the synthesis of a mixture of G1 homo- and heterodendrimers

Scheme S5. Synthesis of G1 dendrimers

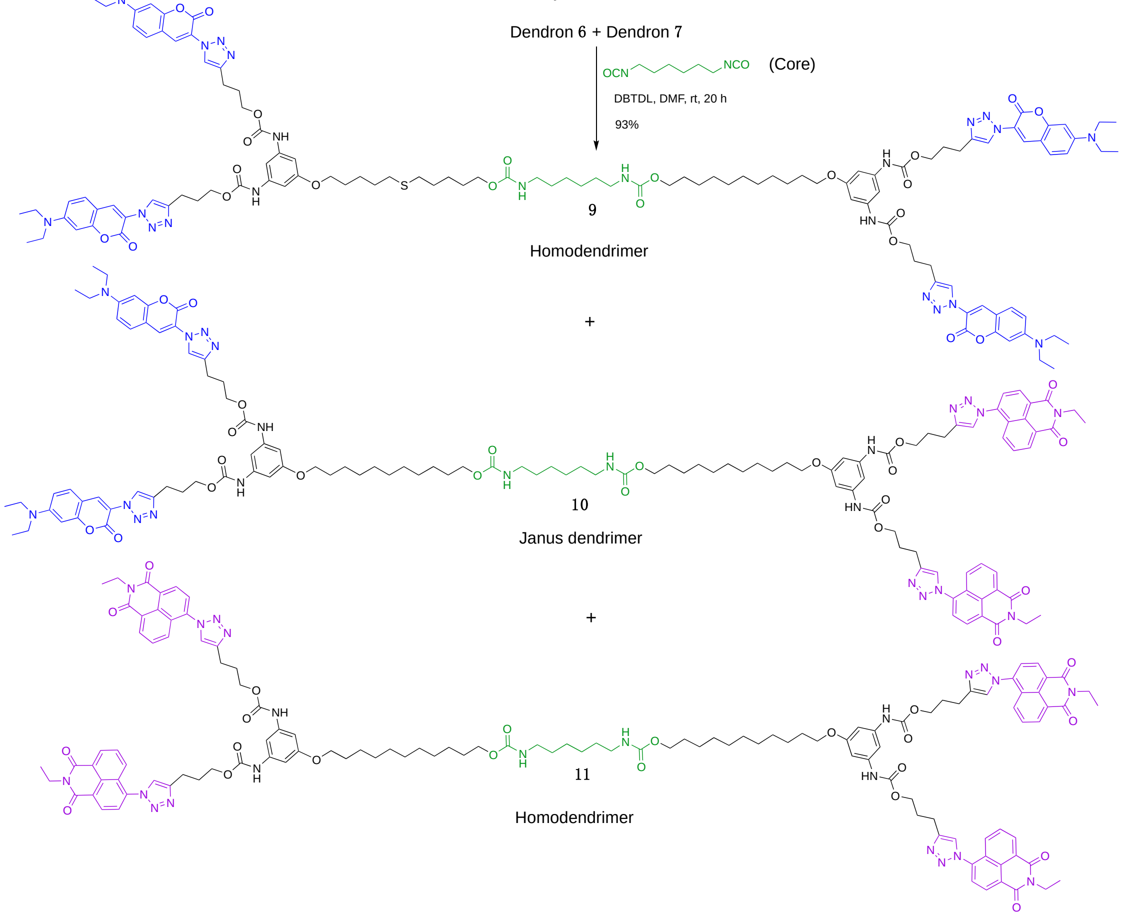

f. Equations showing growth of dendrons 12-16

Scheme S6. Growth of dendrons

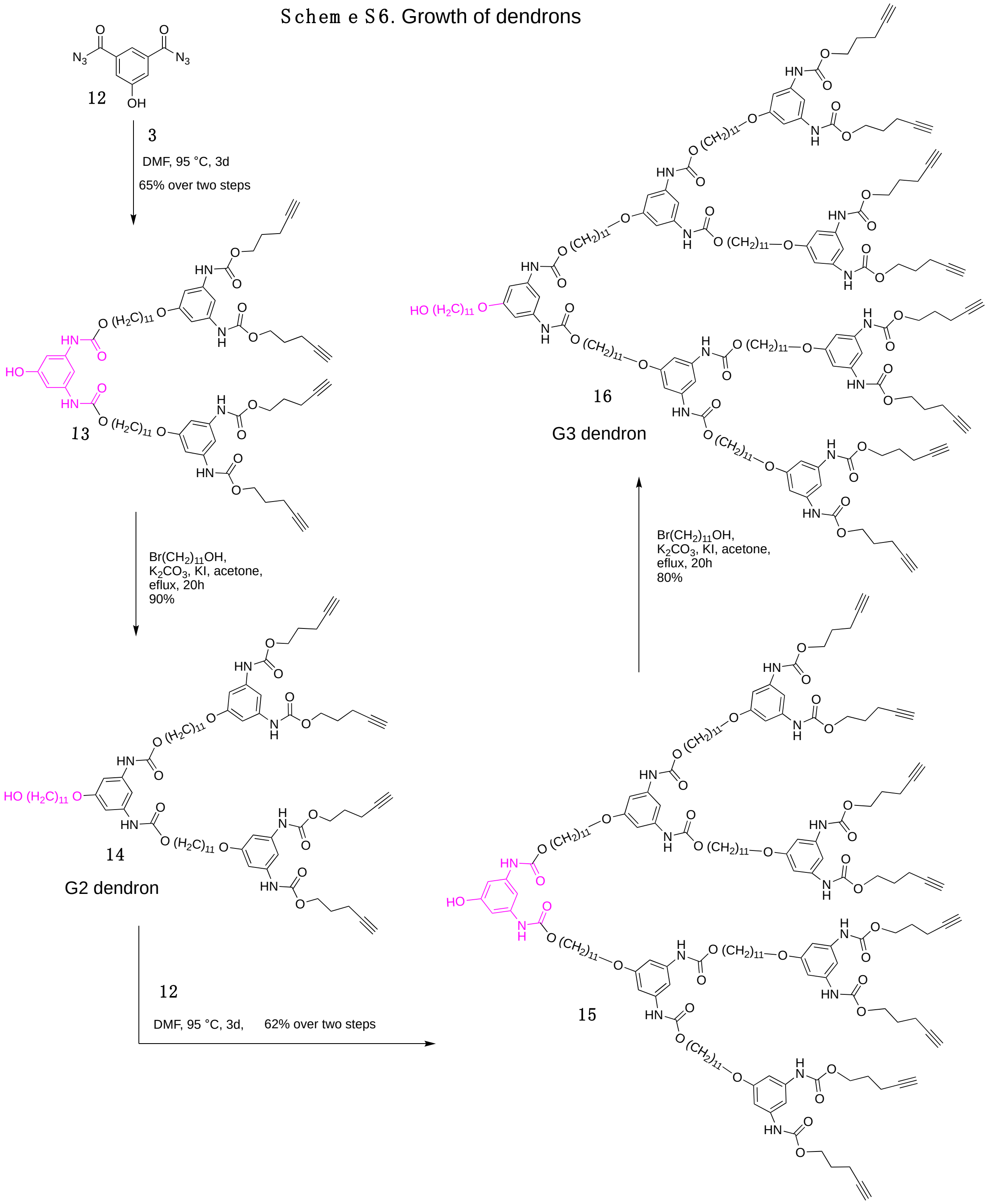

g. Equation showing late-stage modification of polyurethane dendrons 14 and 16

Scheme S7. Late-stage modification of polyurethane dendrons

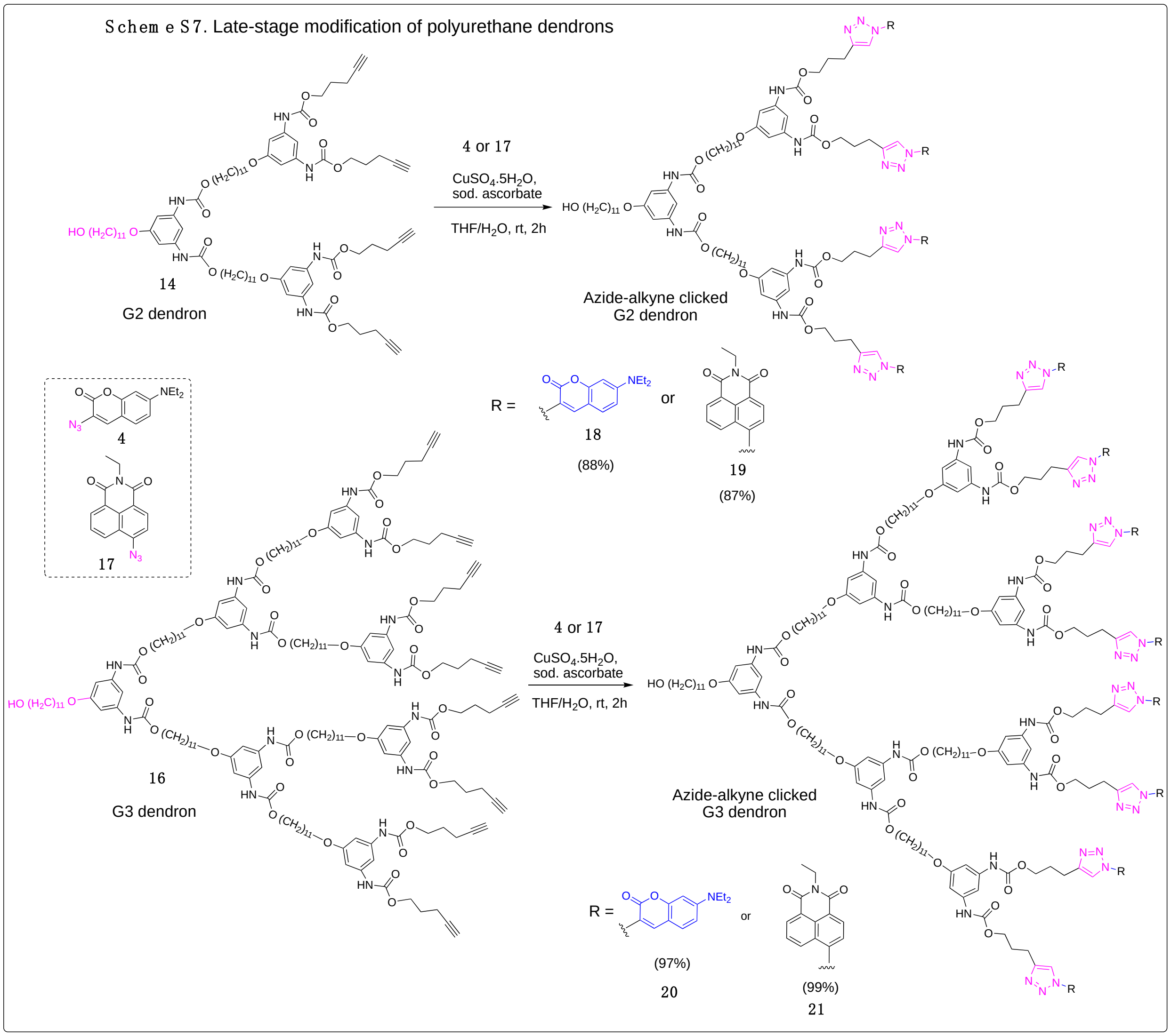

h. Equation for the synthesis of G2 dendrimers 24-26

Scheme S8. Synthesis of G2 homo- and Janus dendrimers

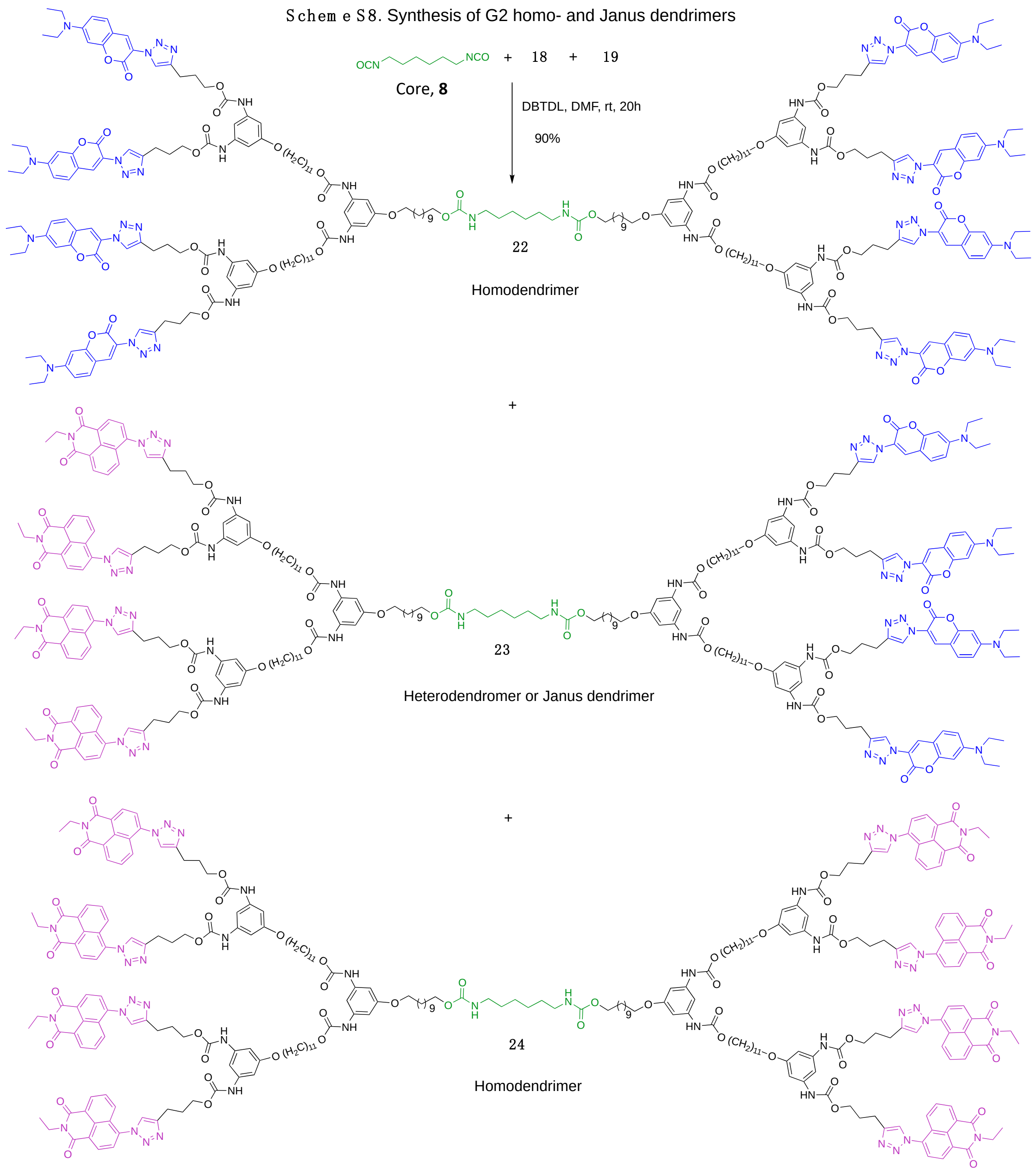

i. Equation for the synthesis of G3 dendrimers and their expanded structures

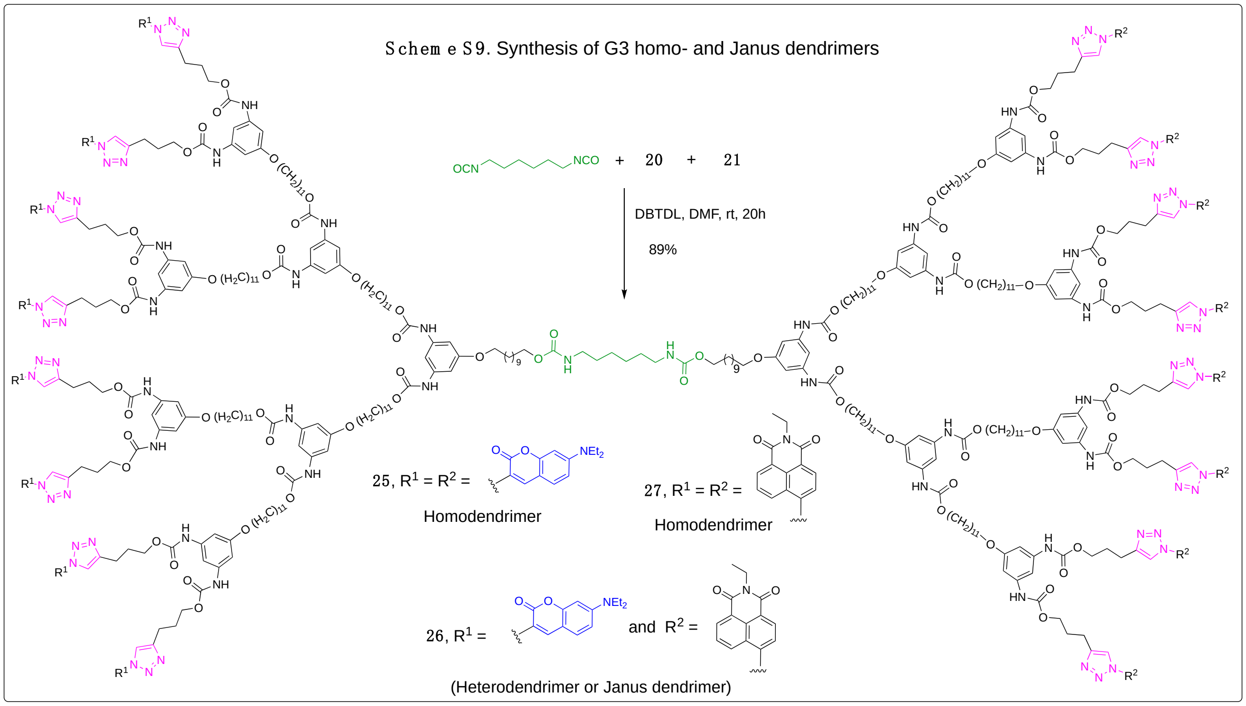

Scheme S9. Synthesis of G3 homo- and Janus dendrimers

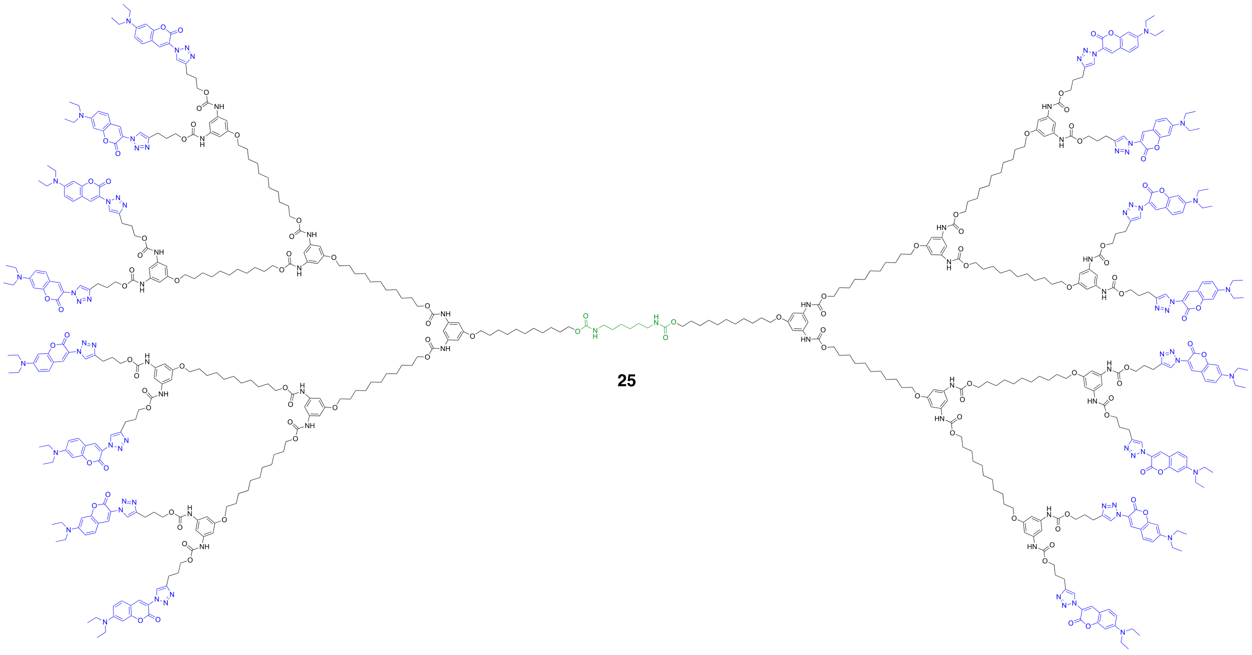

25

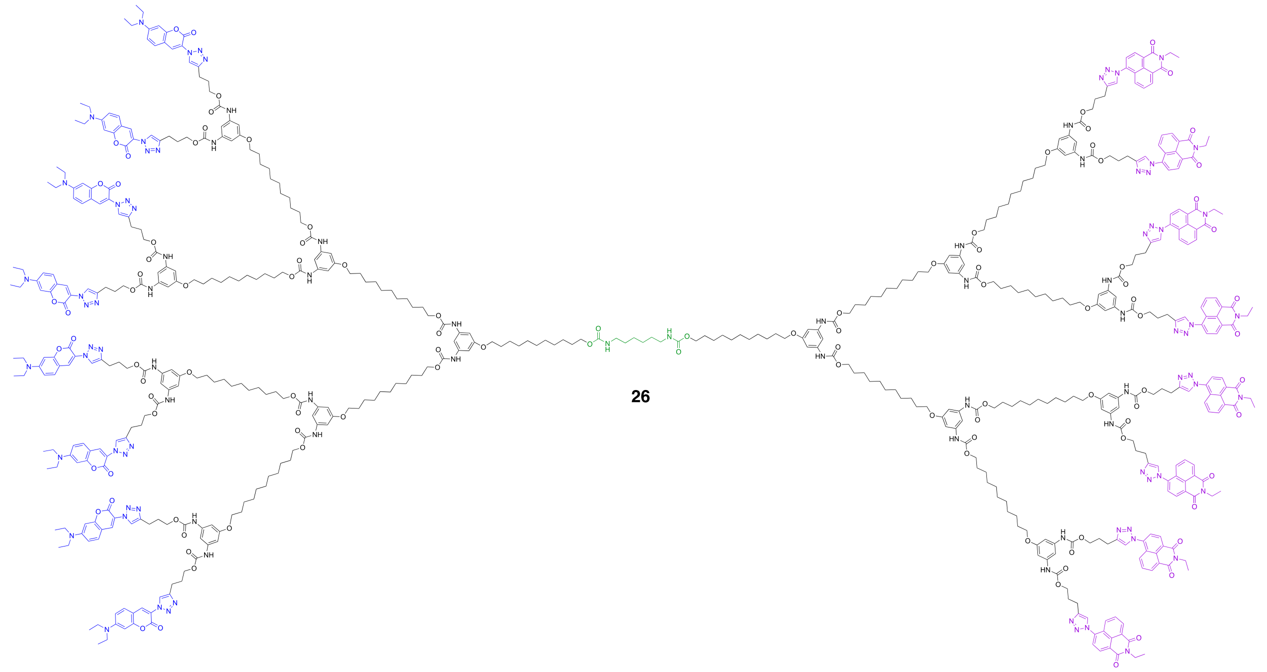

26

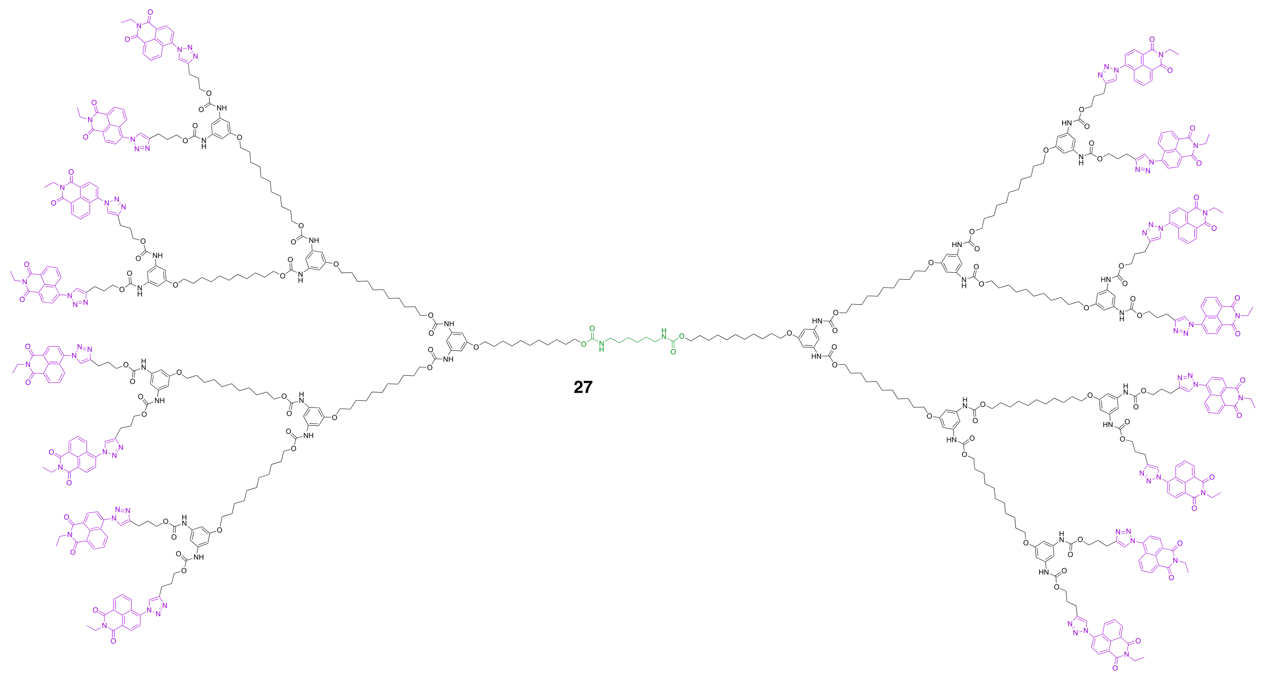

27

S2 Spectral data of polyurethane dendrons and dendrimers

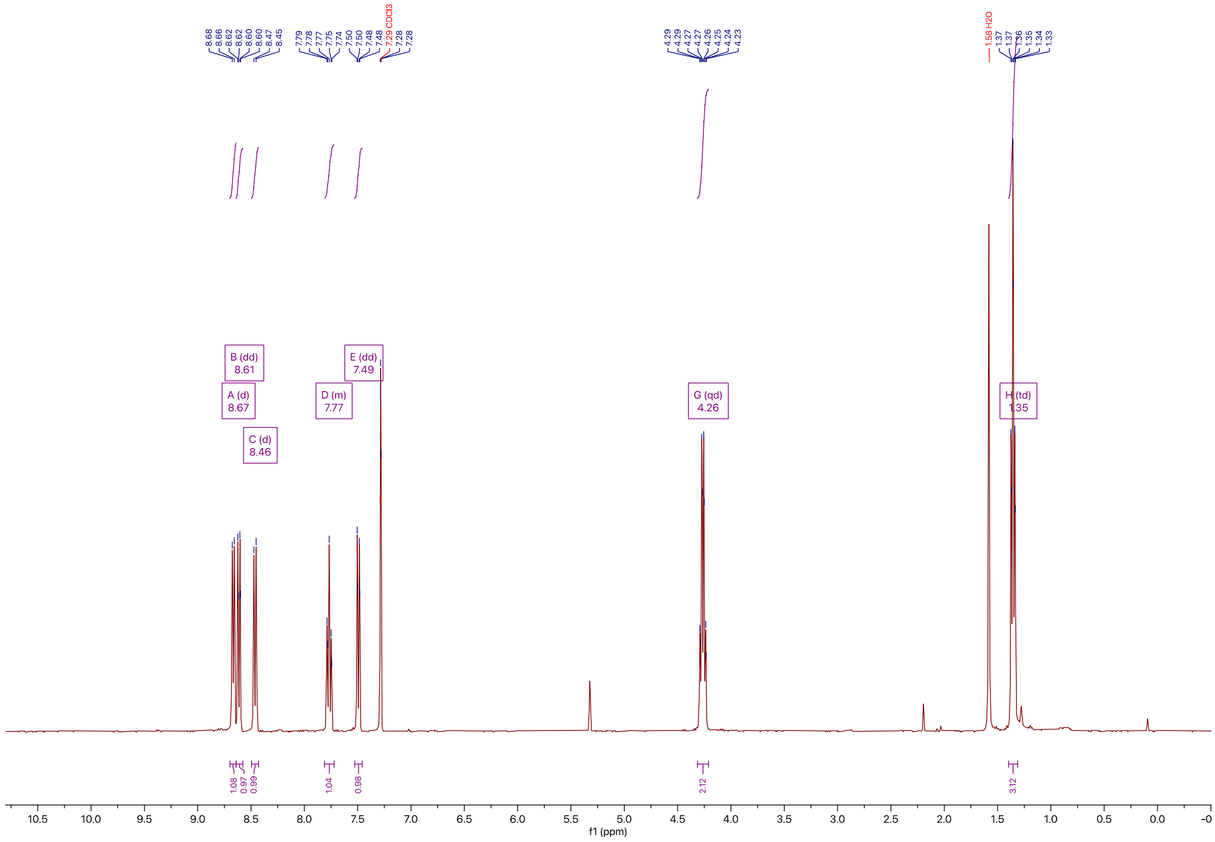

Figure S1. ^{1}H NMR spectrum (400 MHz, CD_3COCD_3, 298 K) of 4-azido-*N*-ethyl-1,8-naphthalimide **5.**

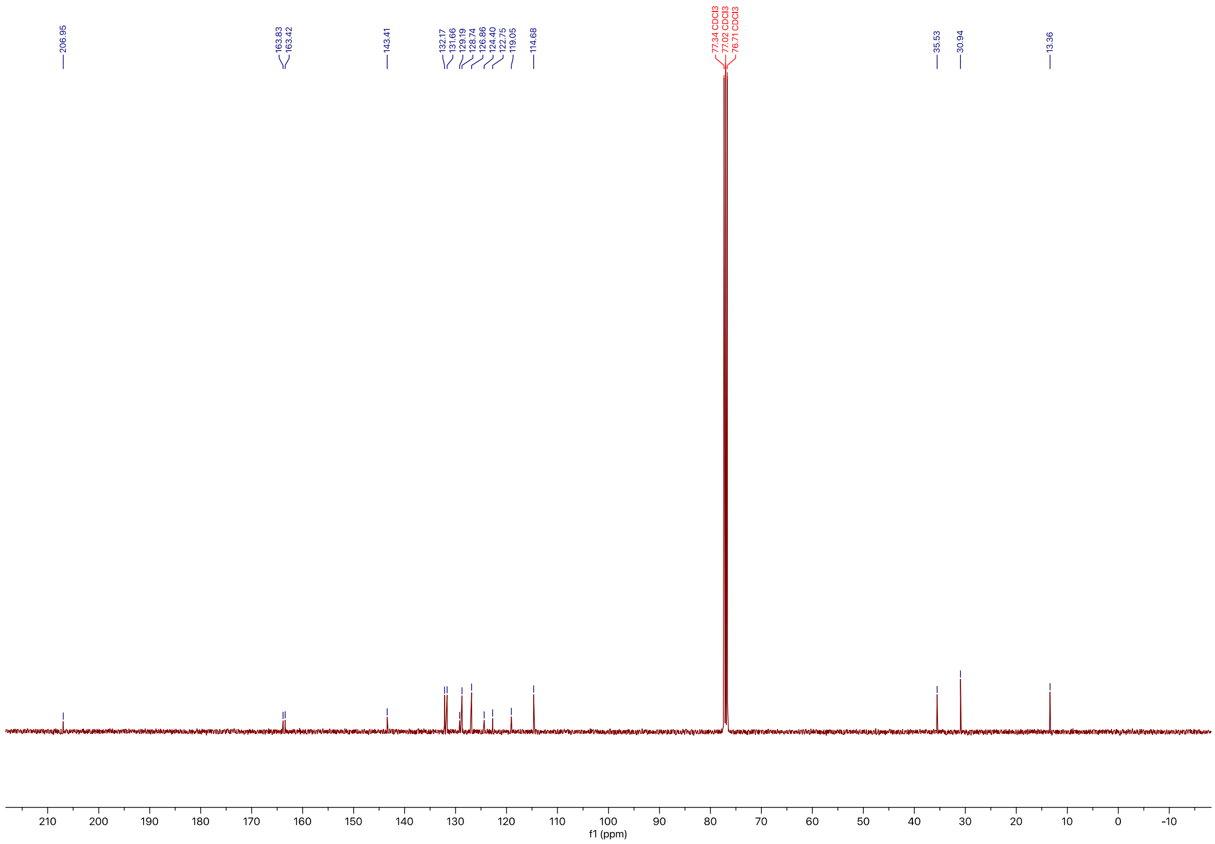

Figure S2. ^{13}C NMR spectrum (101 MHz, CD_3COCD_3, 298 K) of 4-azido-*N*-ethyl-1,8-naphthalimide **5.**

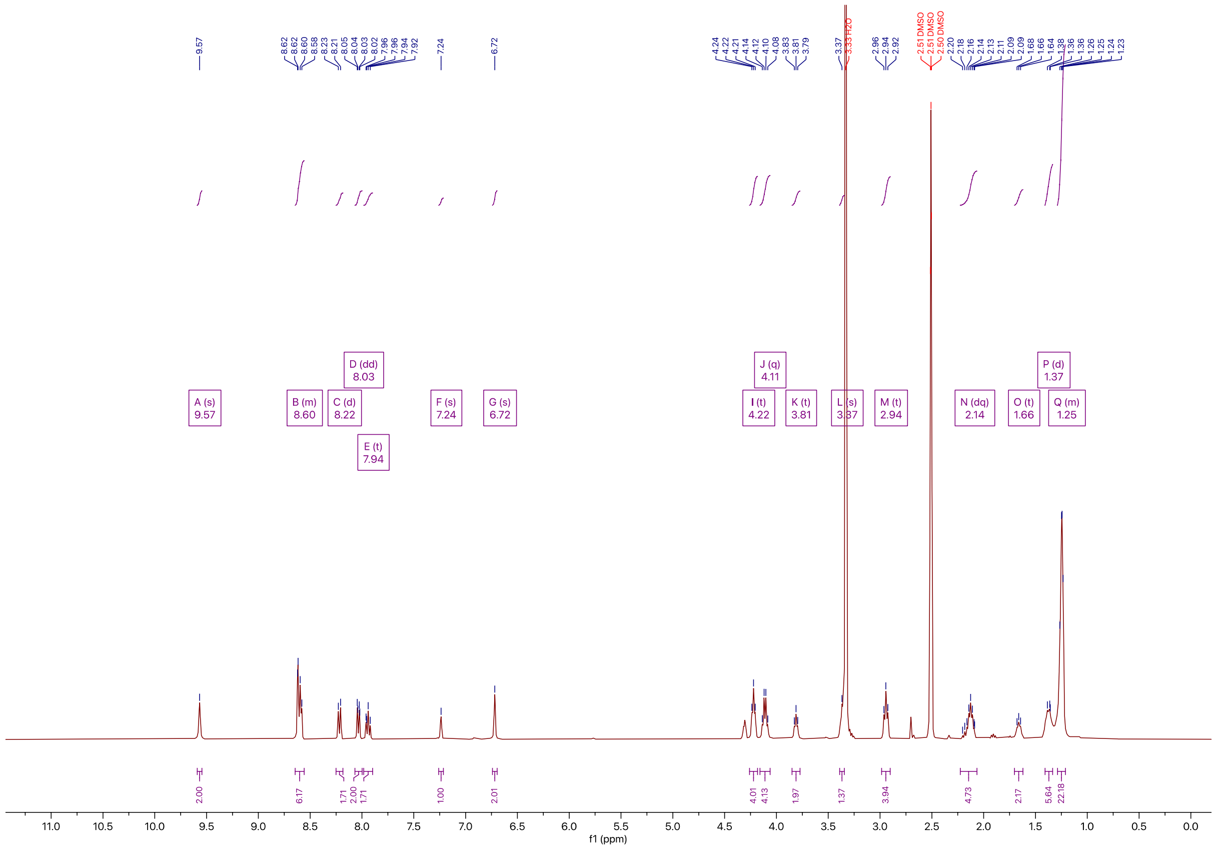

Figure S3. ^{1}H NMR spectrum (500 MHz, CD_3COCD_3, 298 K) of G1 dendron **7**.

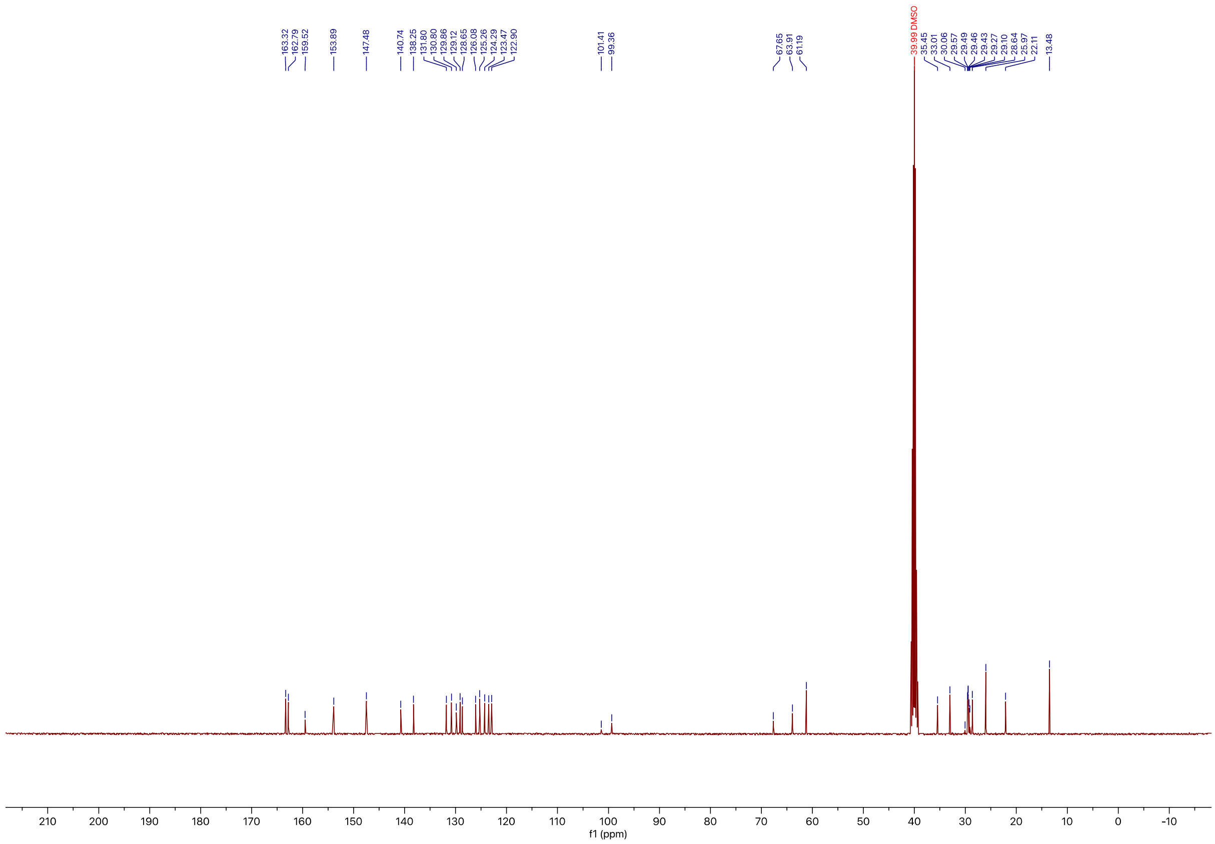

Figure S4. ^{13}C NMR spectrum (126 MHz, CD_3COCD_3, 298 K) of G1 dendron **7**.

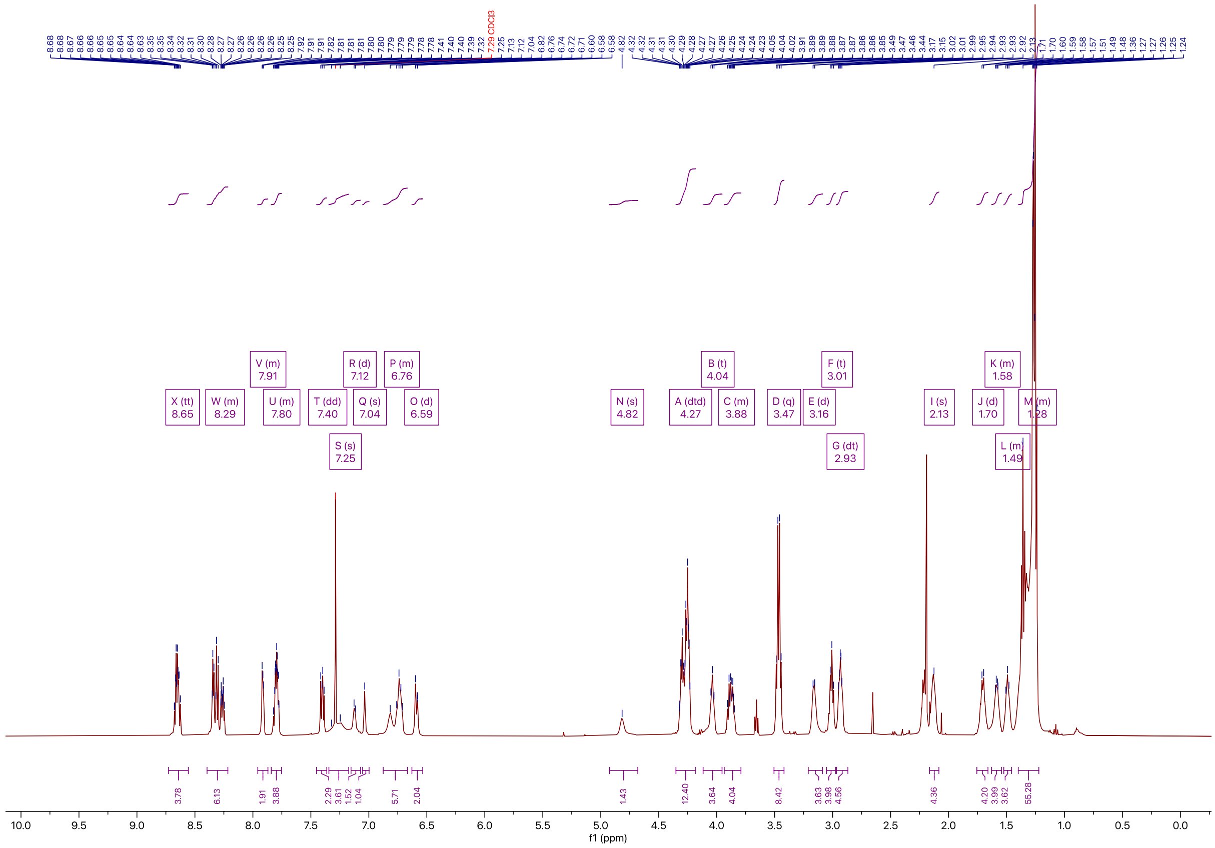

Figure S5. ^{1}H NMR spectrum (500 MHz, $CDCl_3$, 298 K) of G1 Janus dendrimer **10**.

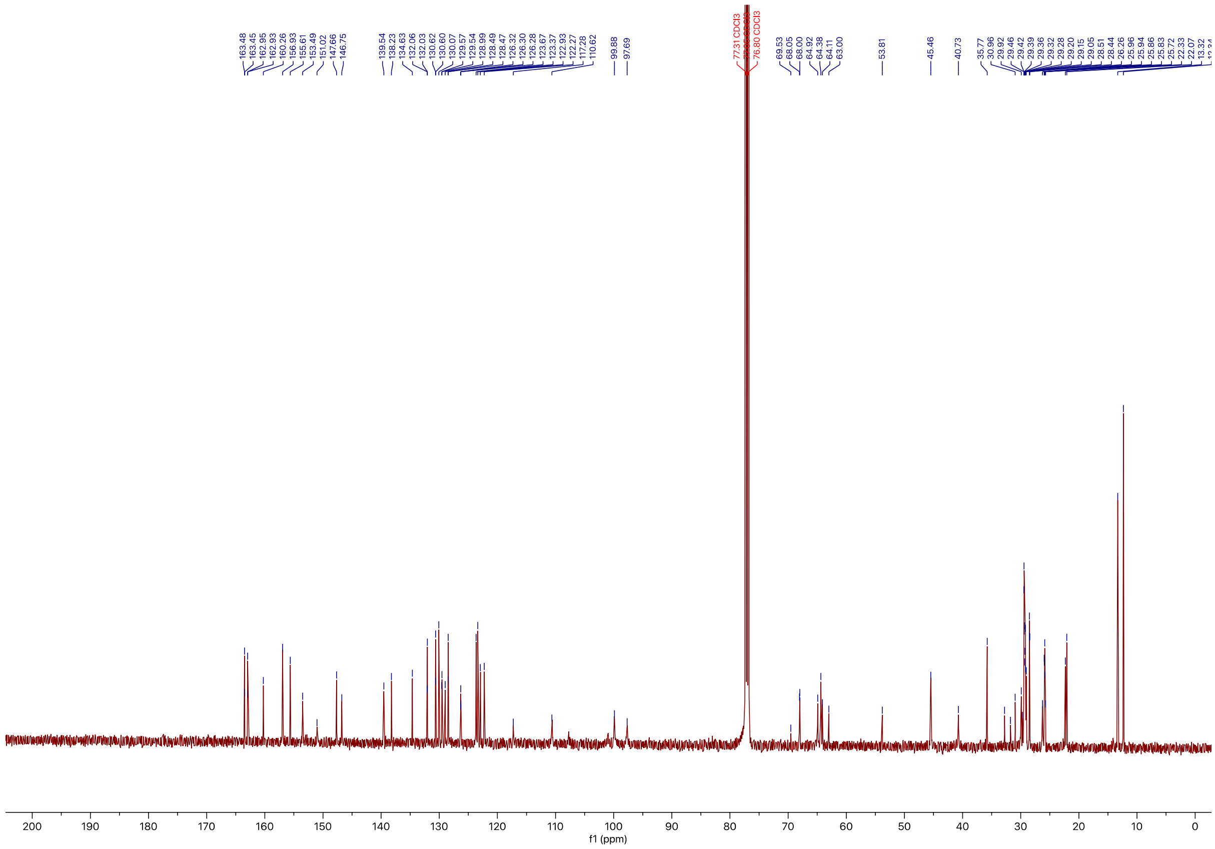

Figure S6. ^{13}C NMR spectrum (126 MHz, $CDCl_3$, 298 K) of G1 dendrimer **10**.

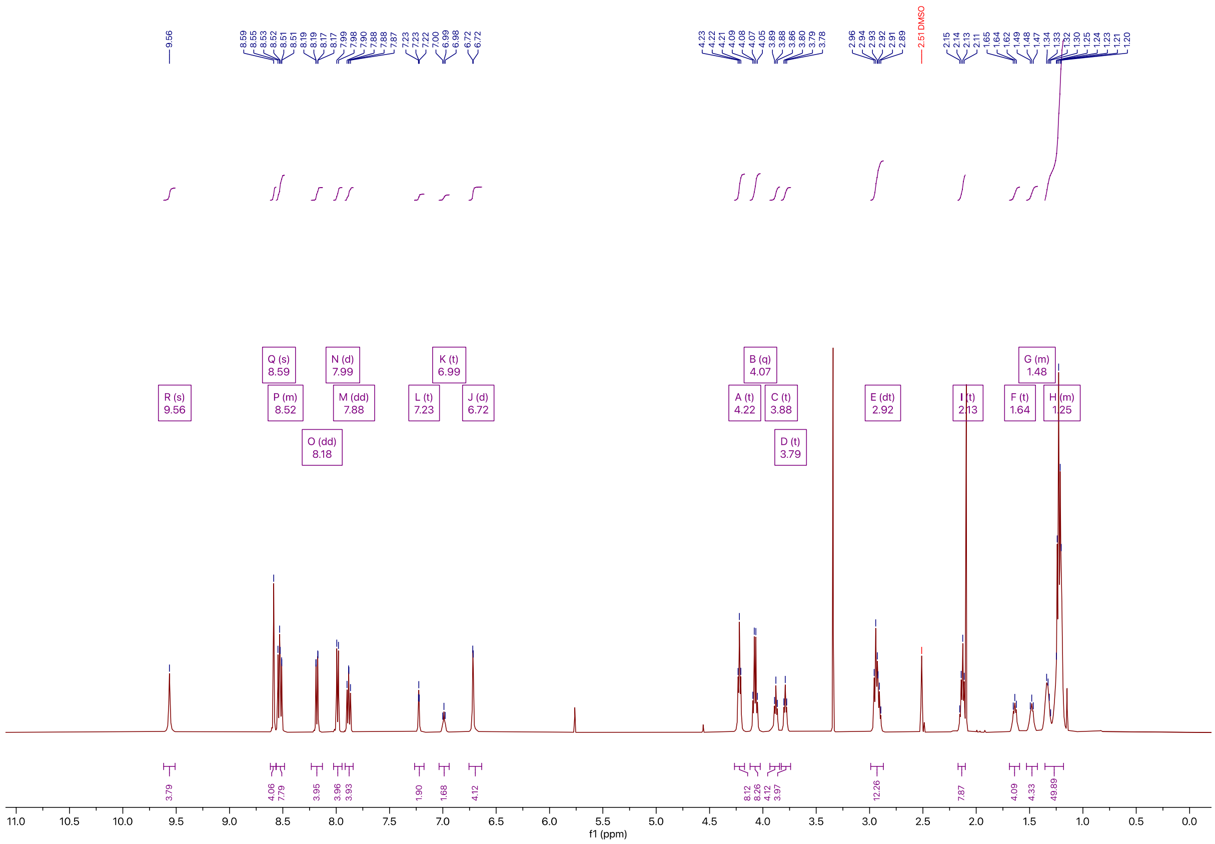

Figure S7. ^{1}H NMR spectrum (500 MHz, DMSO-d_6, 298 K) of G1 dendrimer **11**.

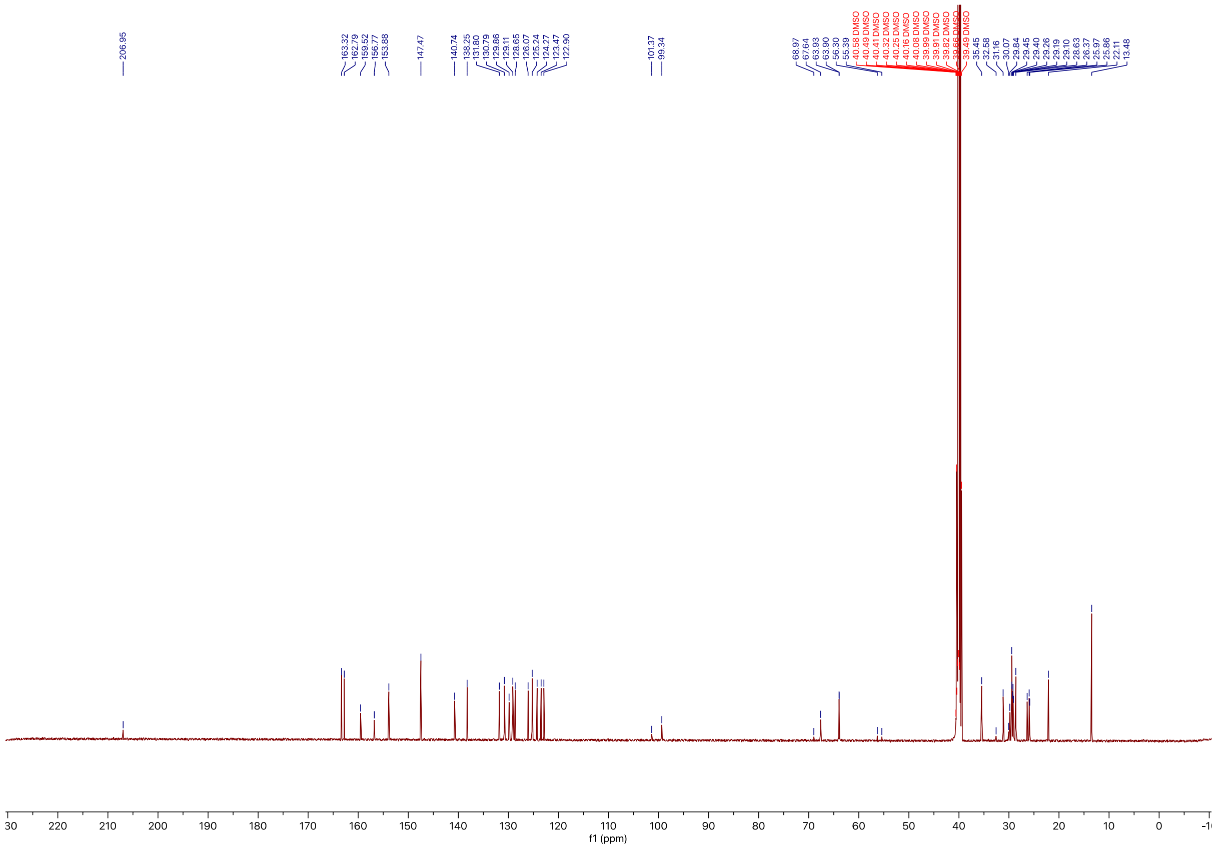

Figure S8. ^{13}C NMR spectrum (126 MHz, DMSO-d_6, 298 K) of G1 dendrimer **11**.

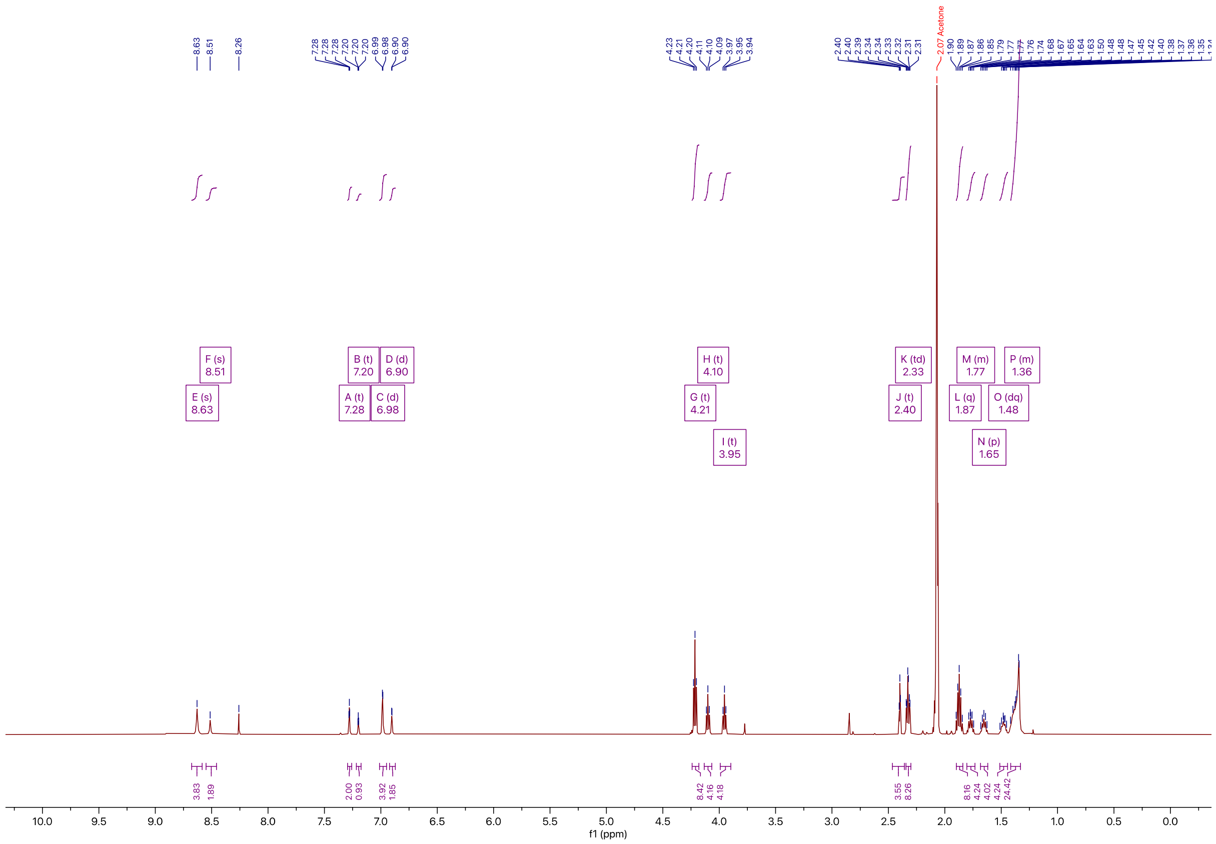

Figure S9. ^{1}H NMR spectrum (500 MHz, CD_3COCD_3, 298 K) of G2 phenolic dendron **13**.

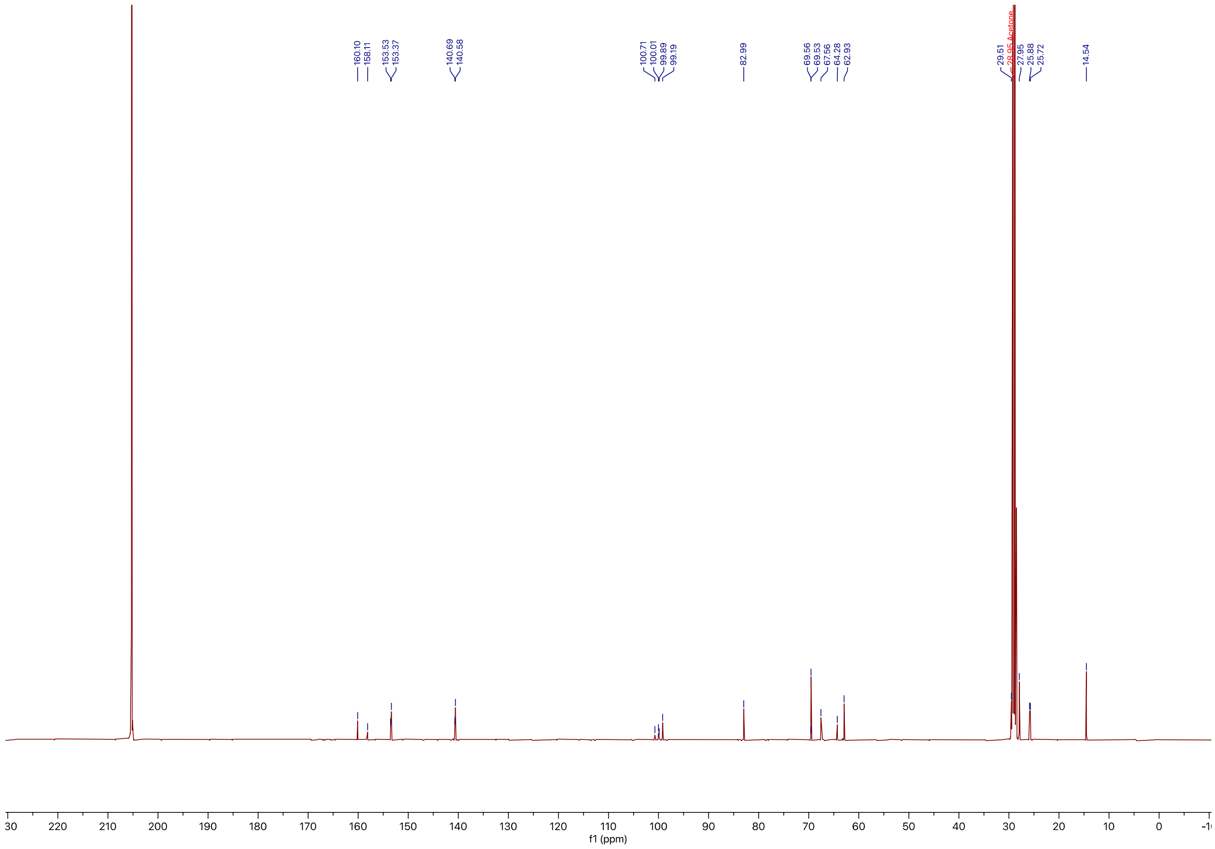

Figure S10. ^{13}C NMR spectrum (126 MHz, CD_3COCD_3, 298 K) of G2 phenolic dendron **13**.

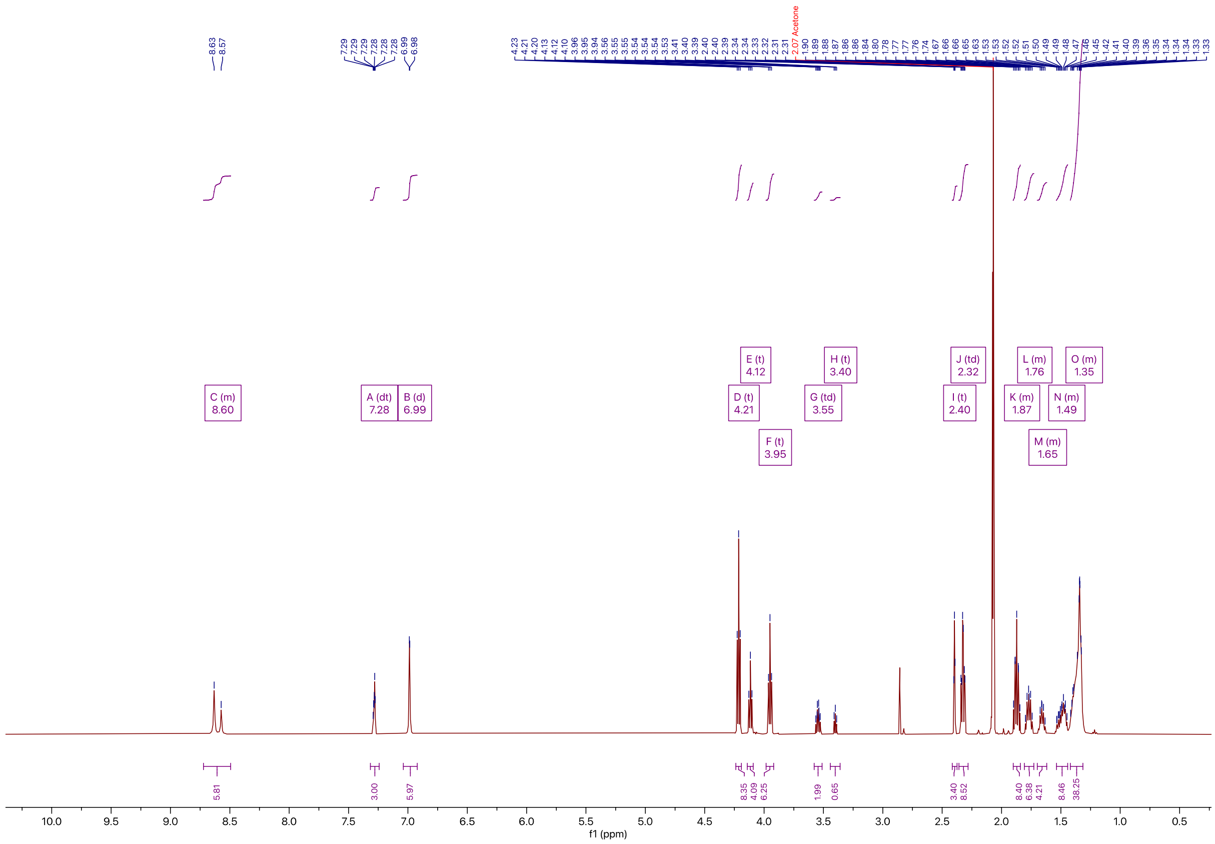

Figure S11. ^{1}H NMR spectrum (500 MHz, CD_3COCD_3, 298 K) of G2 dendron **14**.

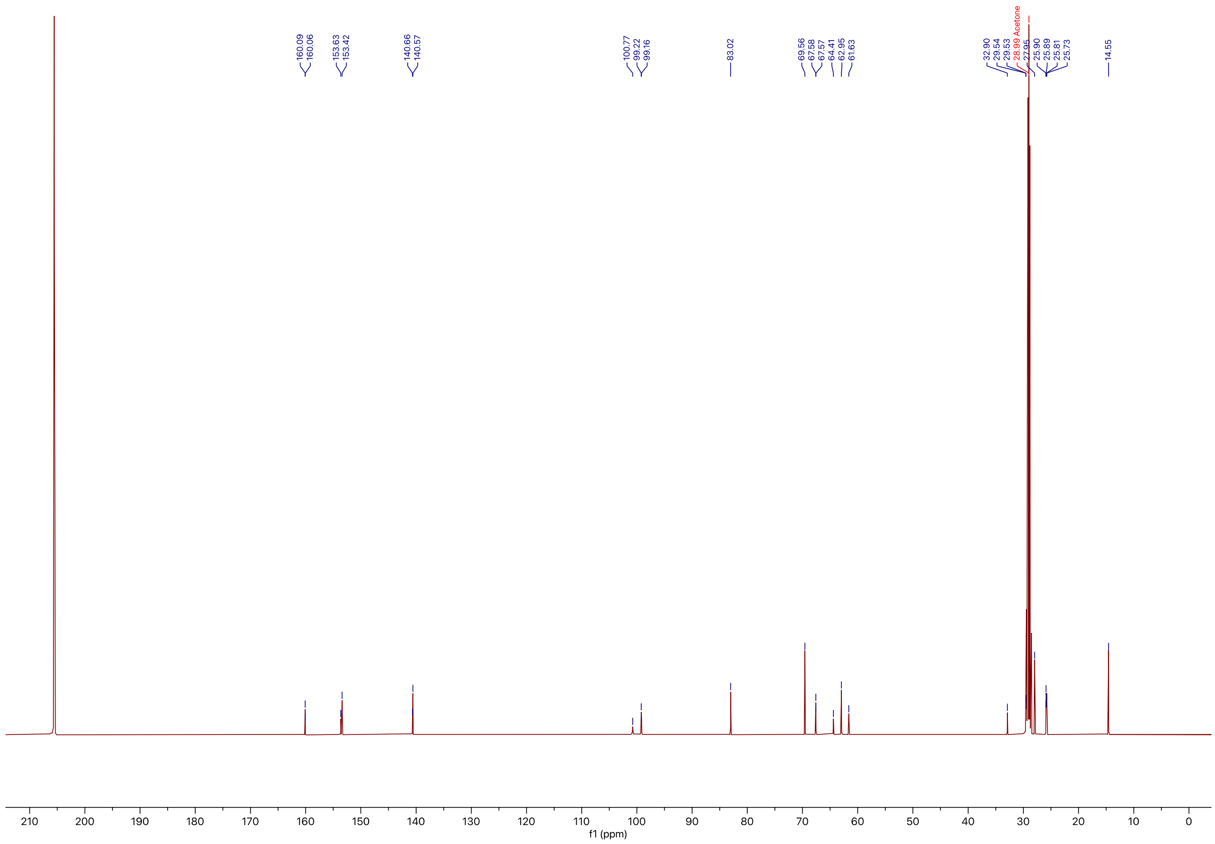

Figure S12. ^{13}C NMR spectrum (126 MHz, CD_3COCD_3, 298 K) of G2 dendron **14**.

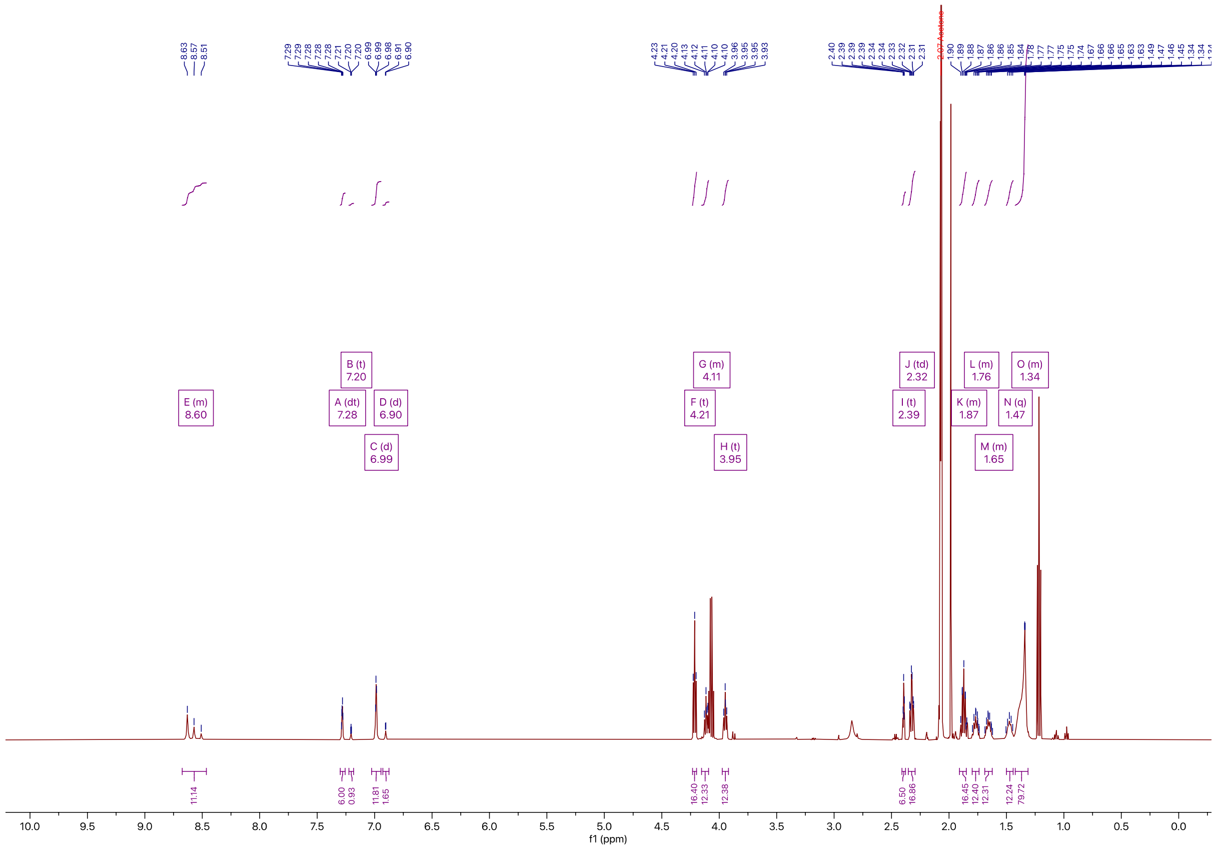

Figure S13. ^{1}H NMR spectrum (500 MHz, CD_3COCD_3, 298 K) of G3 phenolic dendron **15**.

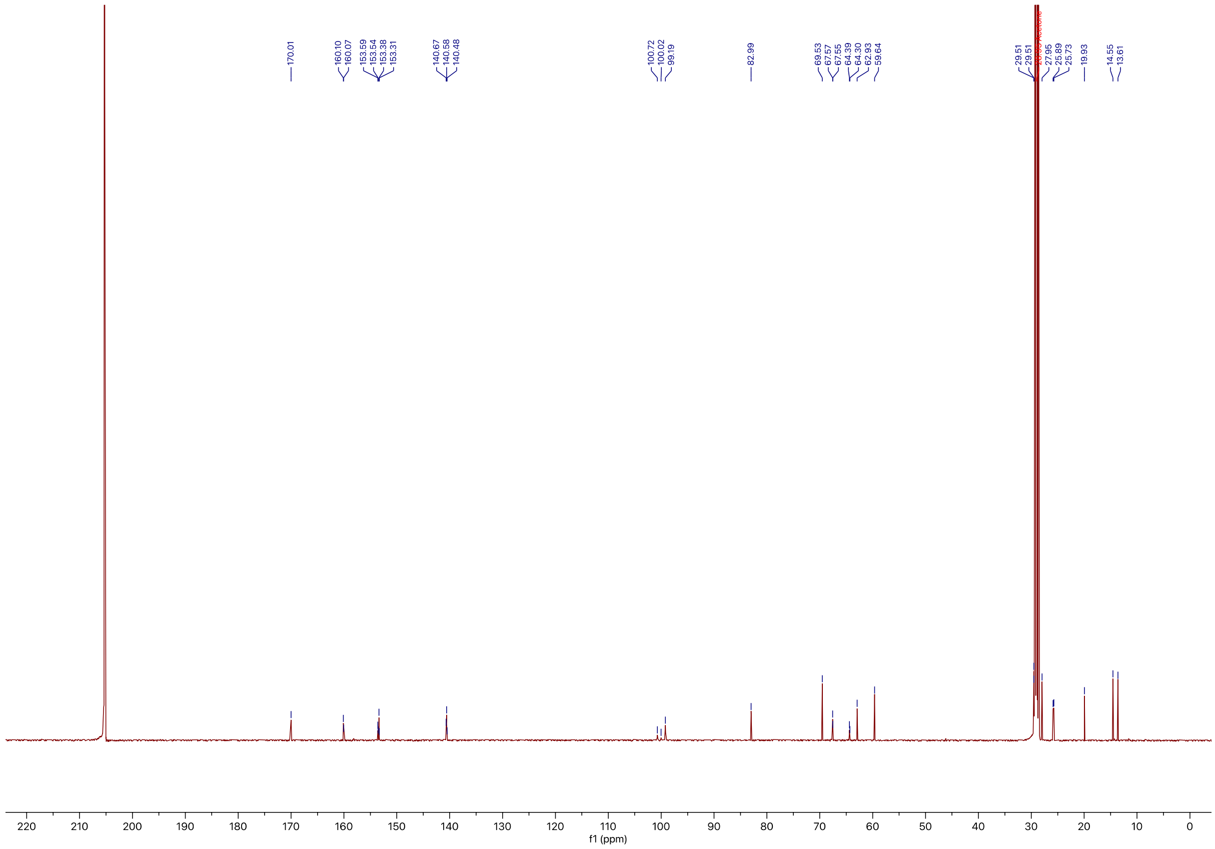

Figure S14. ^{13}C NMR spectrum (126 MHz, CD_3COCD_3, 298 K) of G3 phenolic dendron **15**.

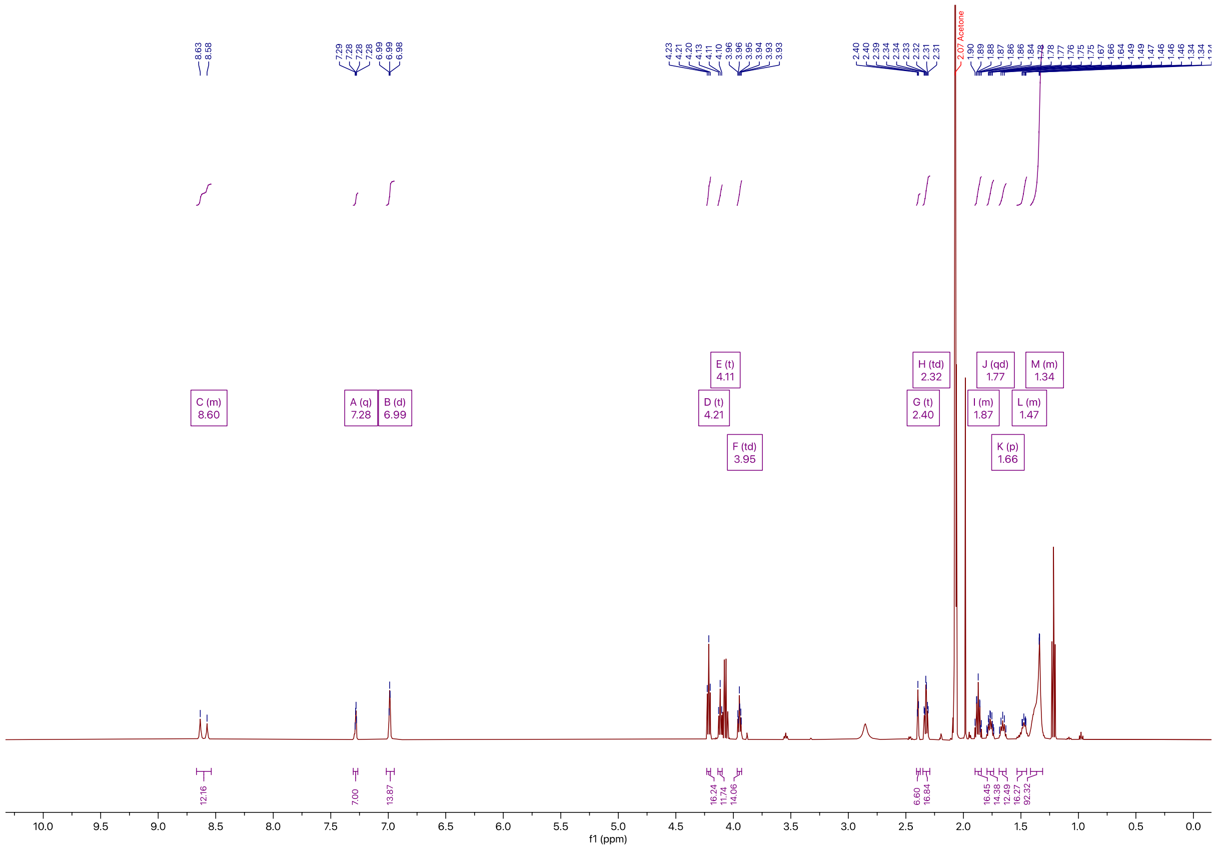

Figure S15. ^{1}H NMR spectrum (500 MHz, CD_3COCD_3, 298 K) of G3 dendron **16**.

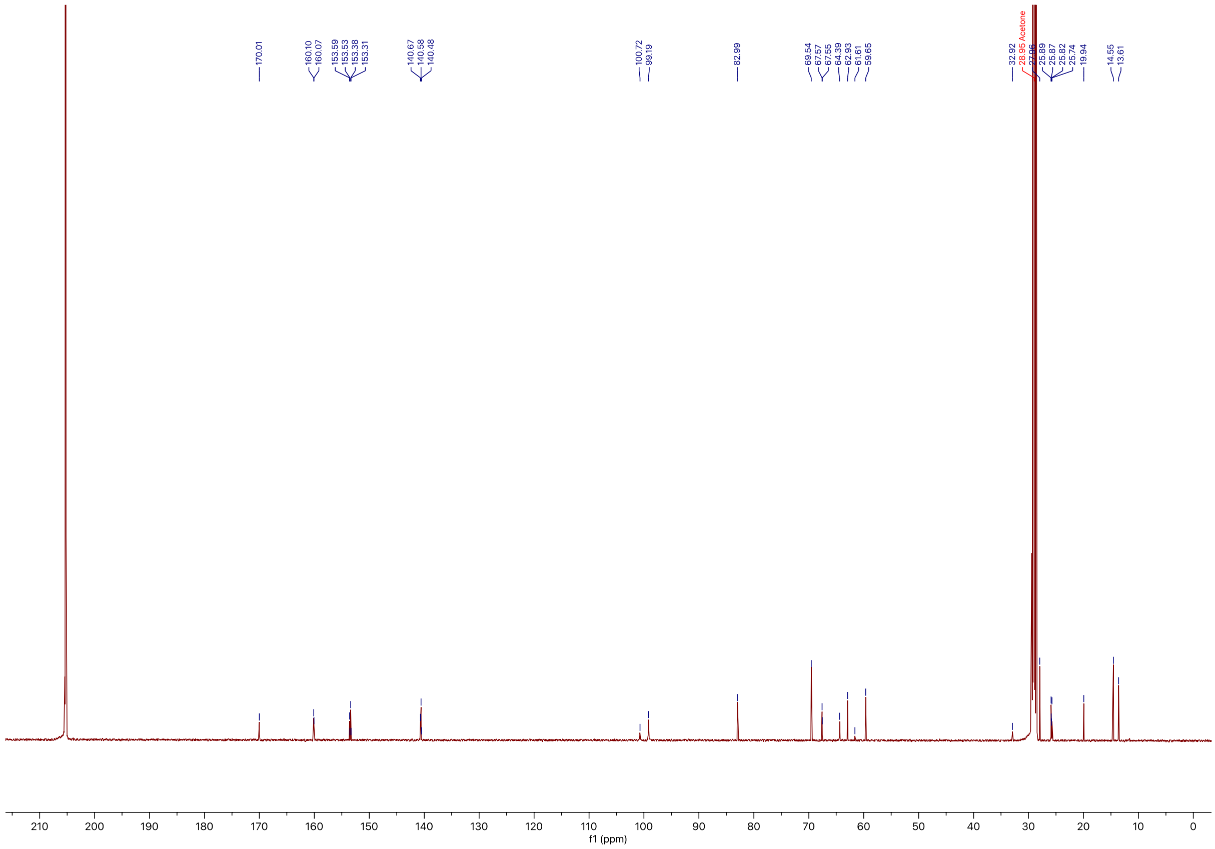

Figure S16. ^{13}C NMR spectrum (126 MHz, CD_3COCD_3, 298 K) of G3 dendron **16**.

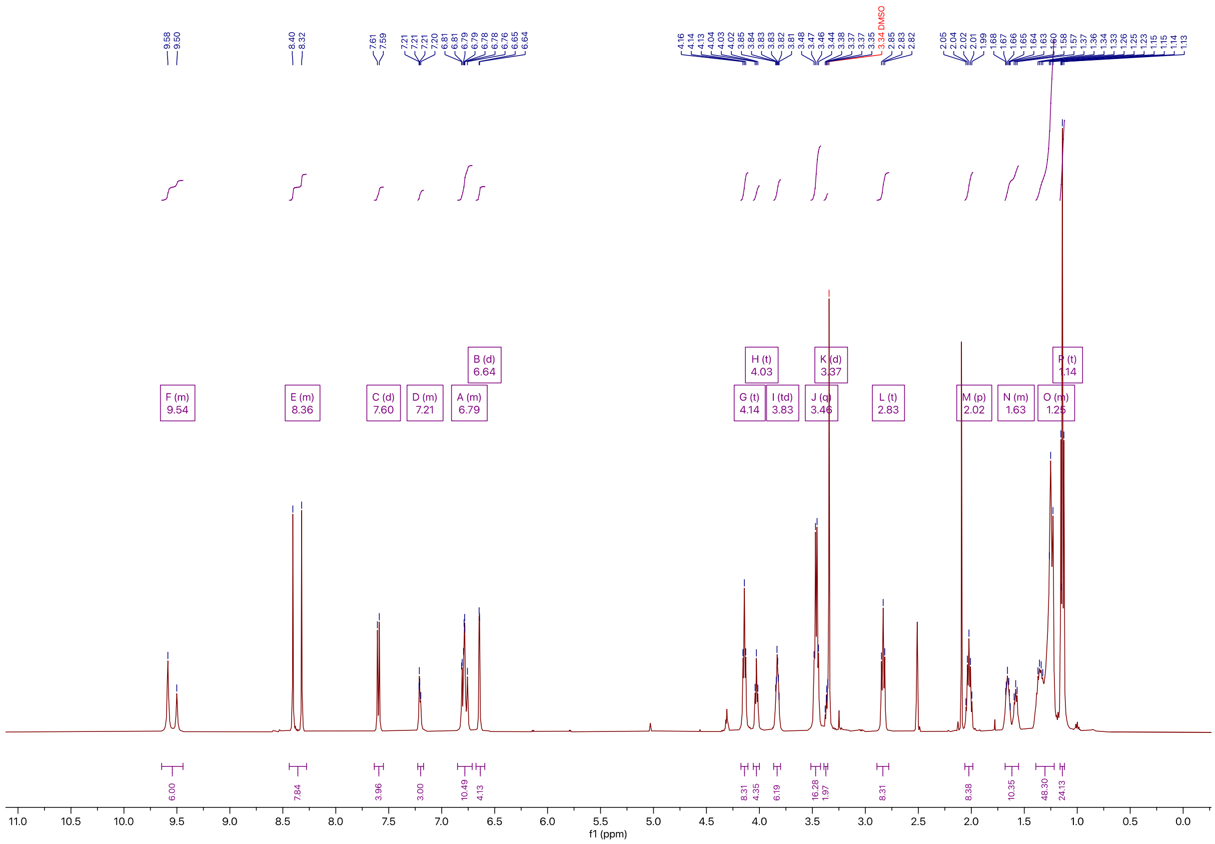

Figure S17. ^{1}H NMR spectrum (500 MHz, DMSO-d_6, 298 K) of blue fluorescent G2 dendron **18**.

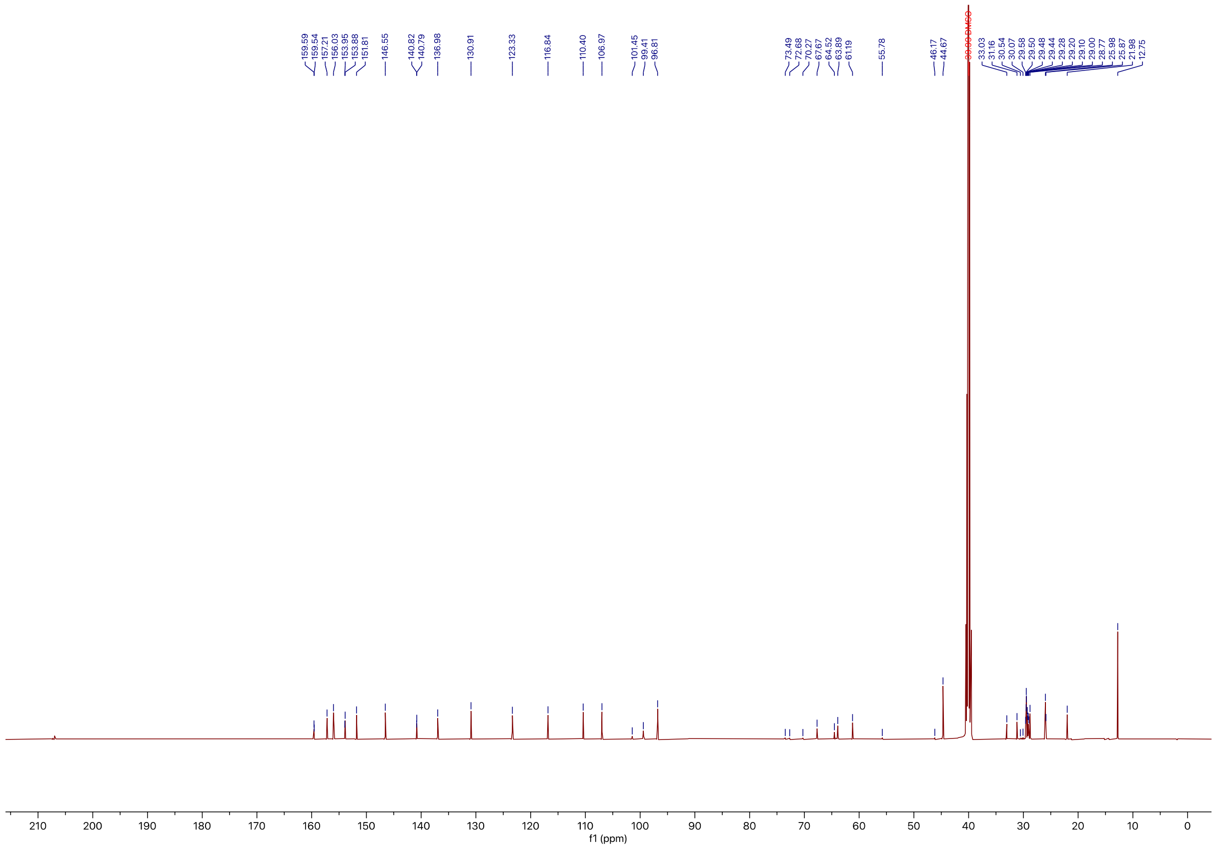

Figure S18. ^{13}C NMR spectrum (126 MHz, DMSO-d_6, 298 K) of blue fluorescent G2 dendron **18**.

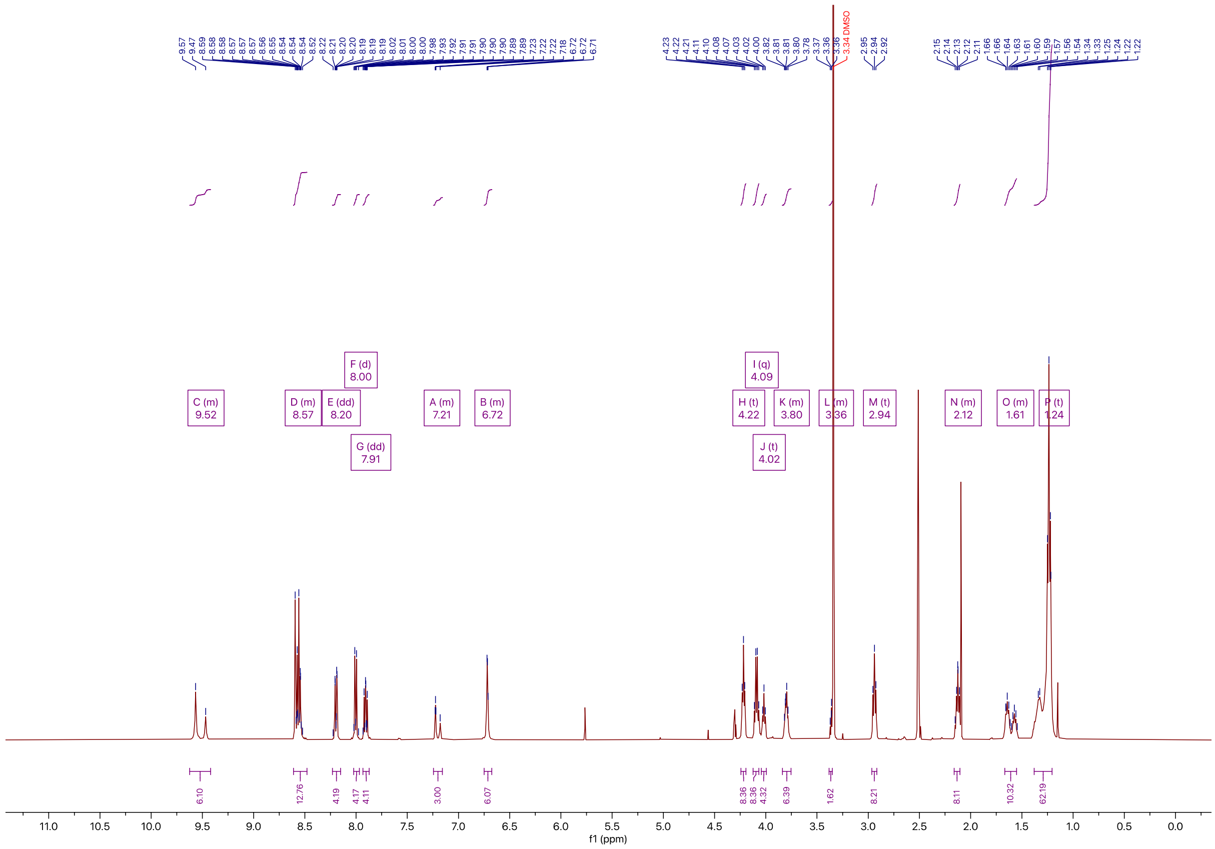

Figure S19. ^{1}H NMR spectrum (500 MHz, DMSO-d_6, 298 K) of naphthalimide-clicked G2 dendron **19**.

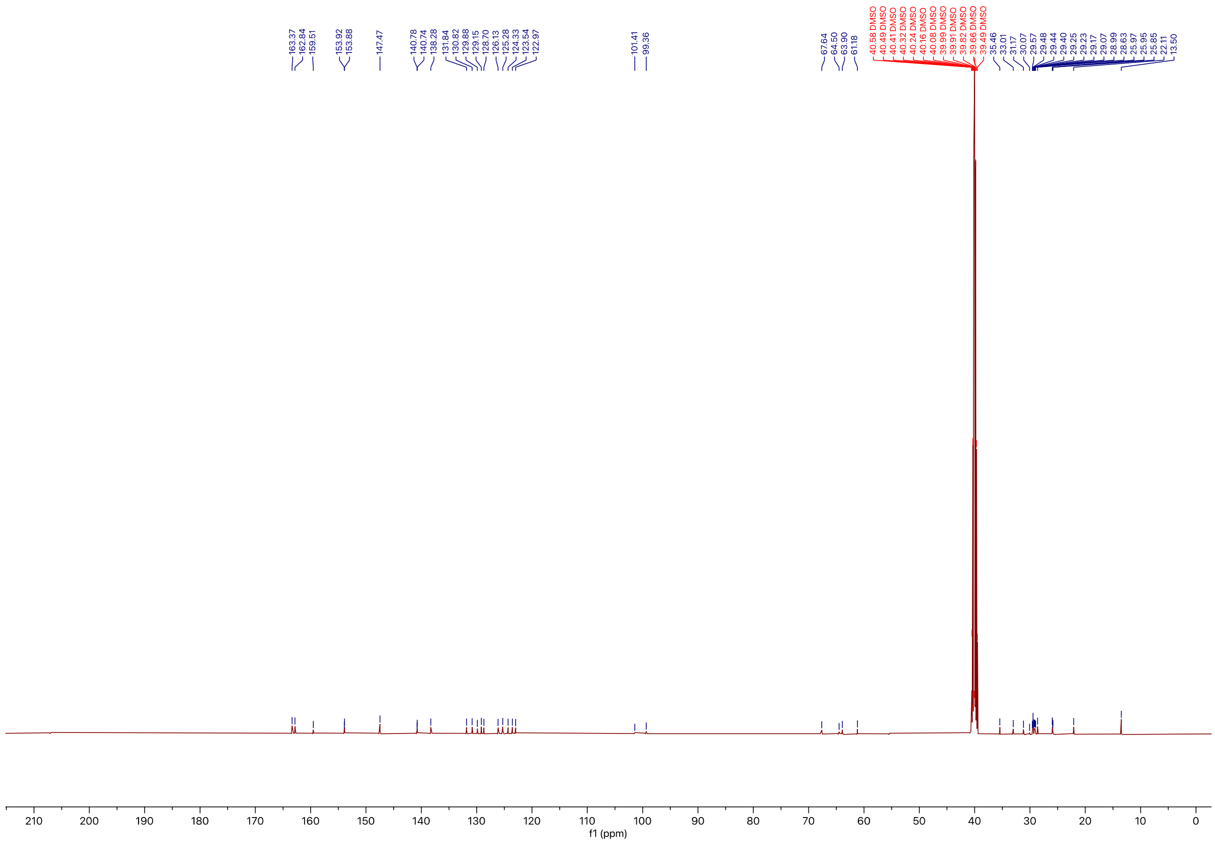

Figure S20. ^{13}C NMR spectrum (126 MHz, DMSO-d_6, 298 K) of naphthalimide-clicked G2 dendron **19**.

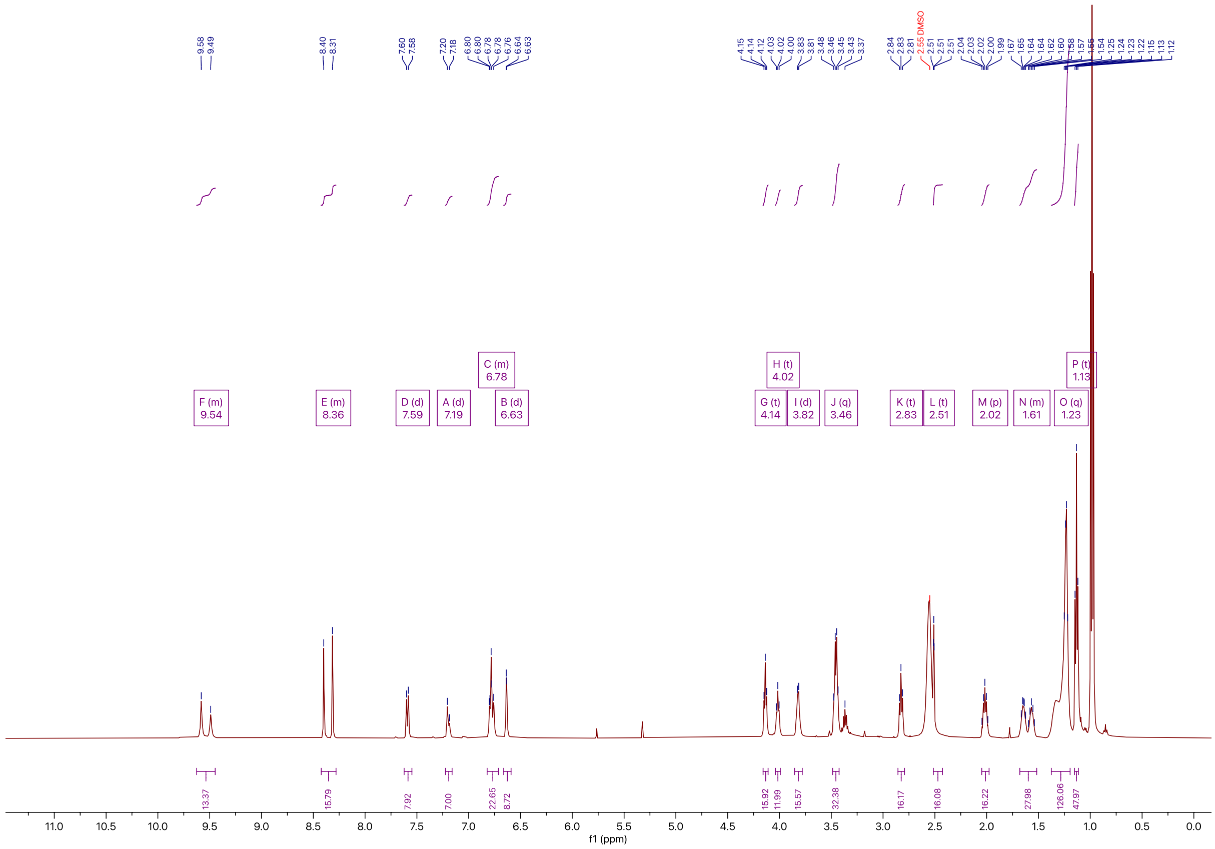

Figure S21. ^{1}H NMR spectrum (500 MHz, DMSO-d_6, 298 K) of blue fluorescent G3 dendron **20**.

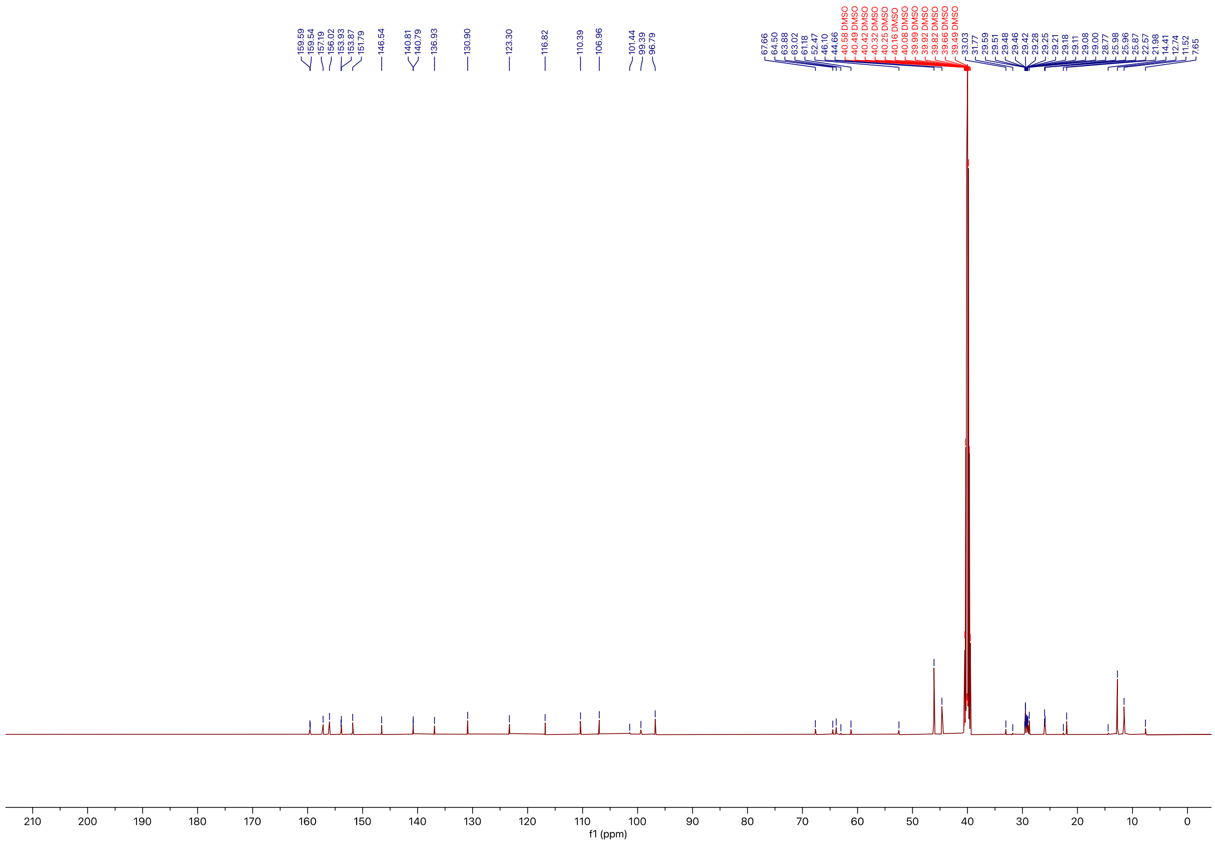

Figure S22. ^{13}C NMR spectrum (126 MHz, DMSO-d_6, 298 K) of blue fluorescent G3 dendron **20**.

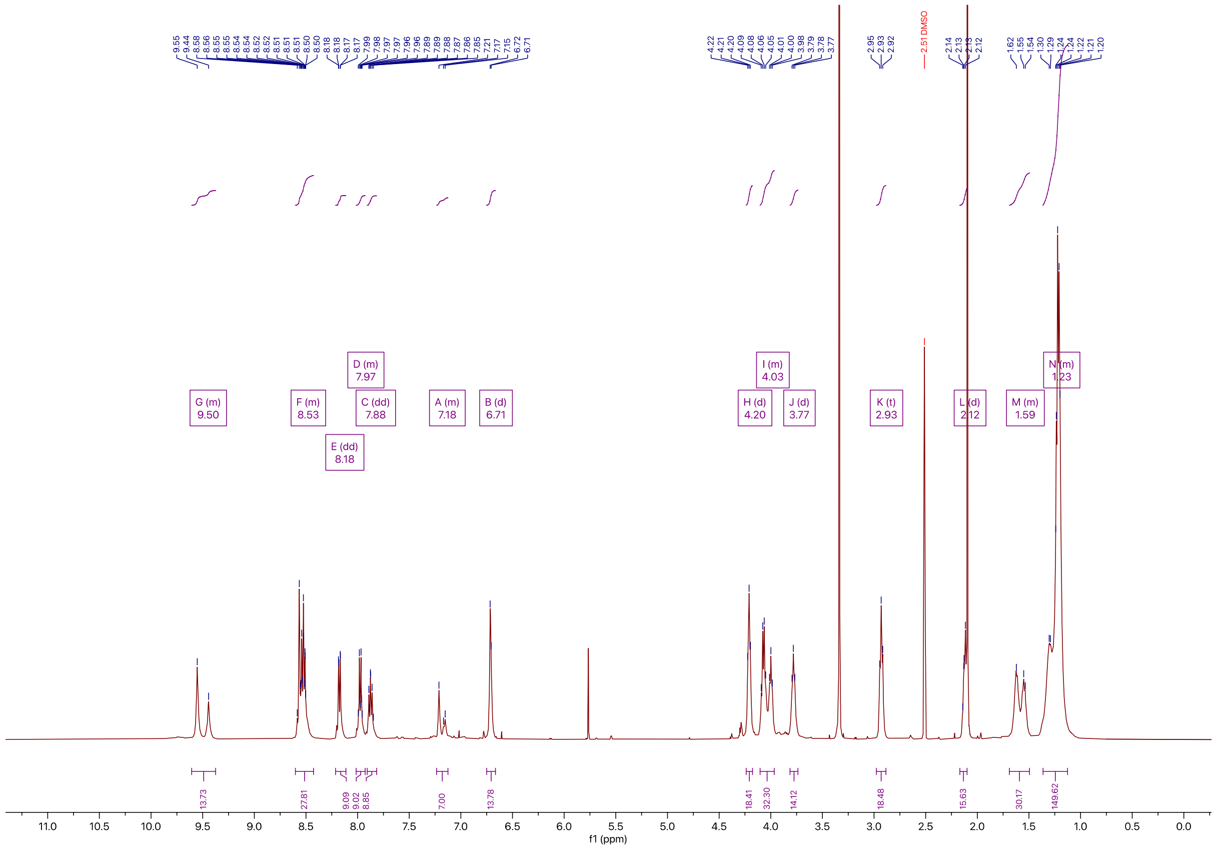

Figure S23. ^{1}H NMR spectrum (500 MHz, DMSO-d_6, 298 K) of naphthalimide-clicked G3 dendron **21**.

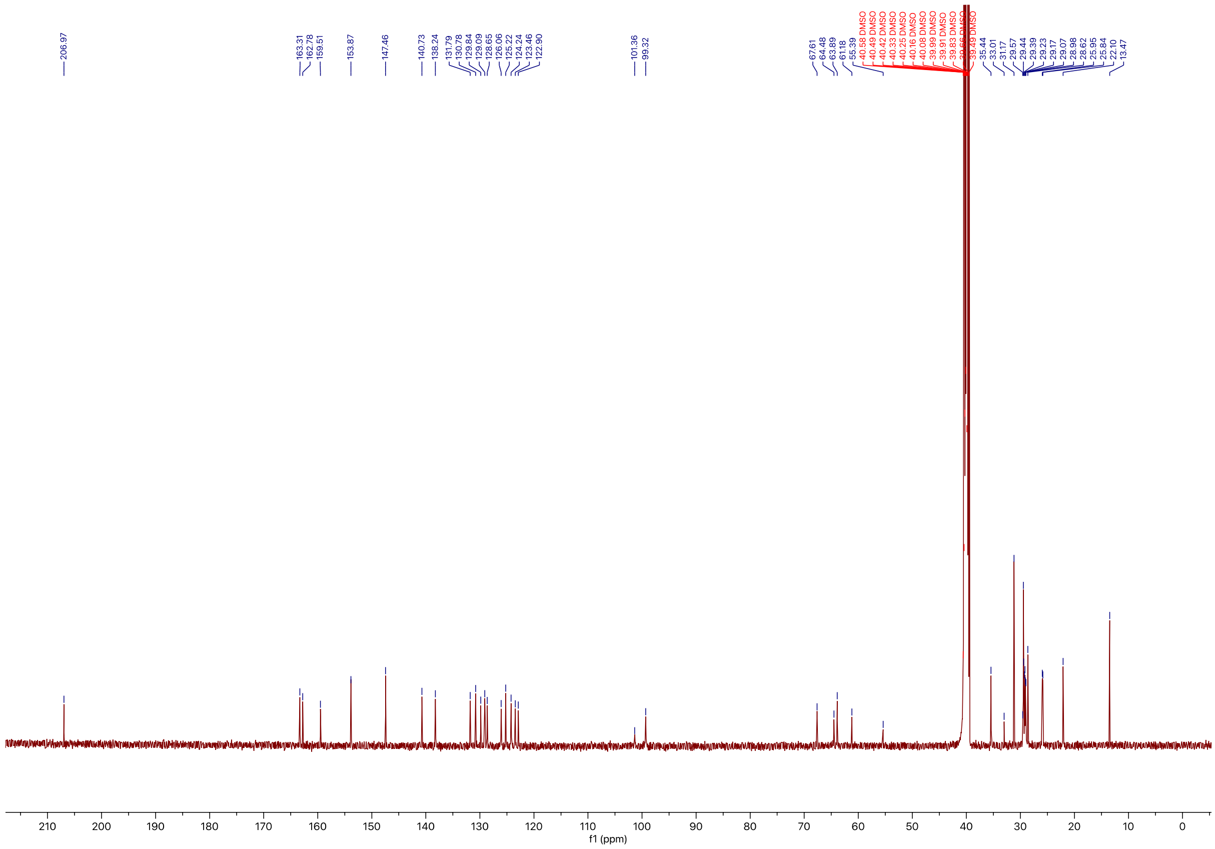

Figure S24. ^{13}C NMR spectrum (126 MHz, DMSO-d_6, 298 K) of naphthalimide-clicked G3 dendron **21**.

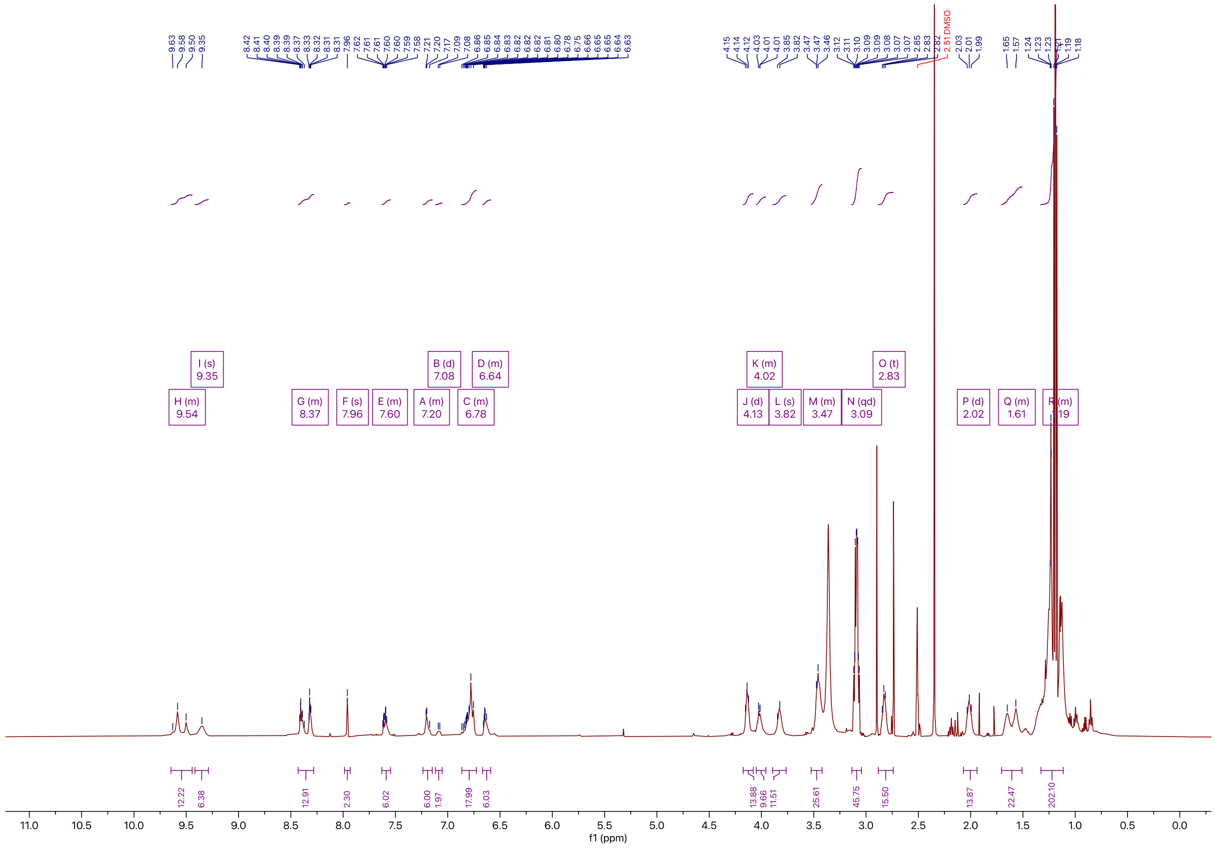

Figure S25. ^{1}H NMR spectrum (500 MHz, DMSO-d_6, 298 K) of blue fluorescent G2 dendrimer **22**.

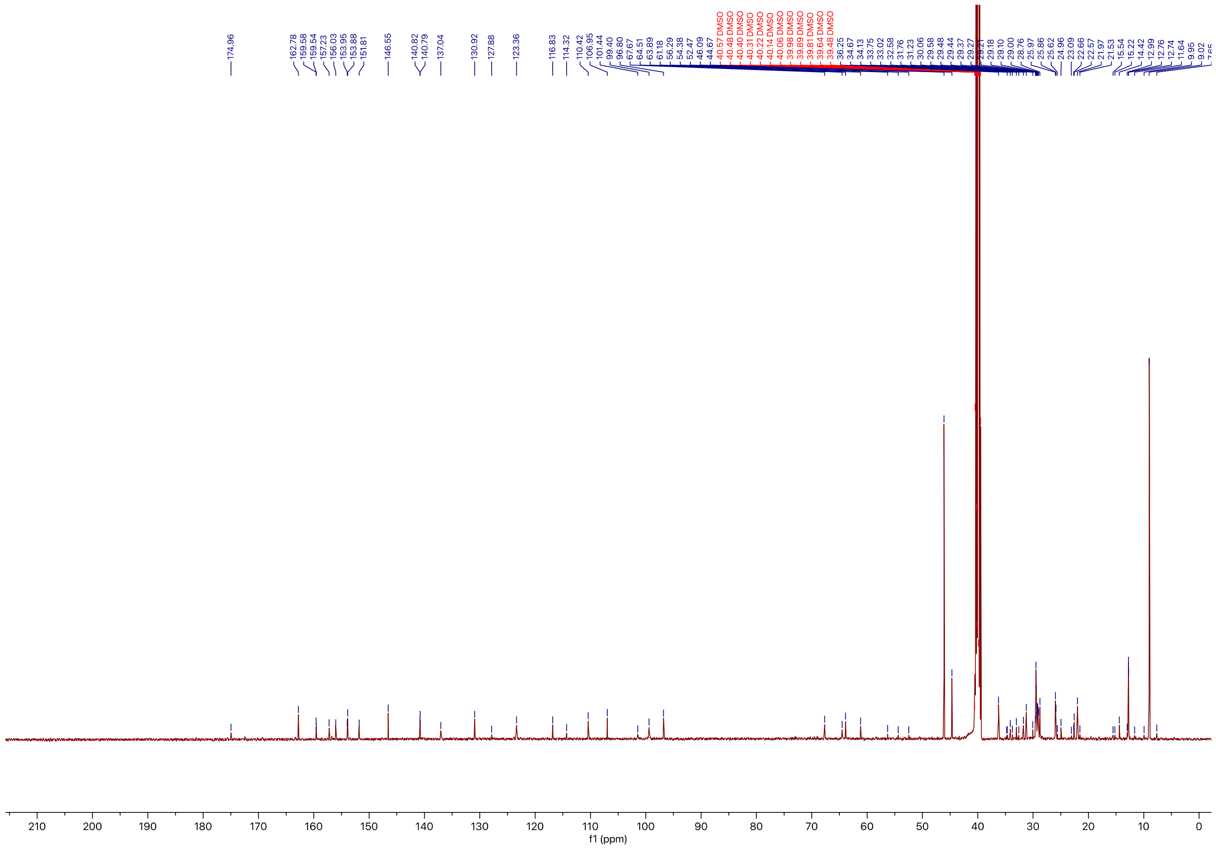

Figure S26. ^{13}C NMR spectrum (126 MHz, DMSO-d_6, 298 K) of blue fluorescent G2 dendrimer **22**.

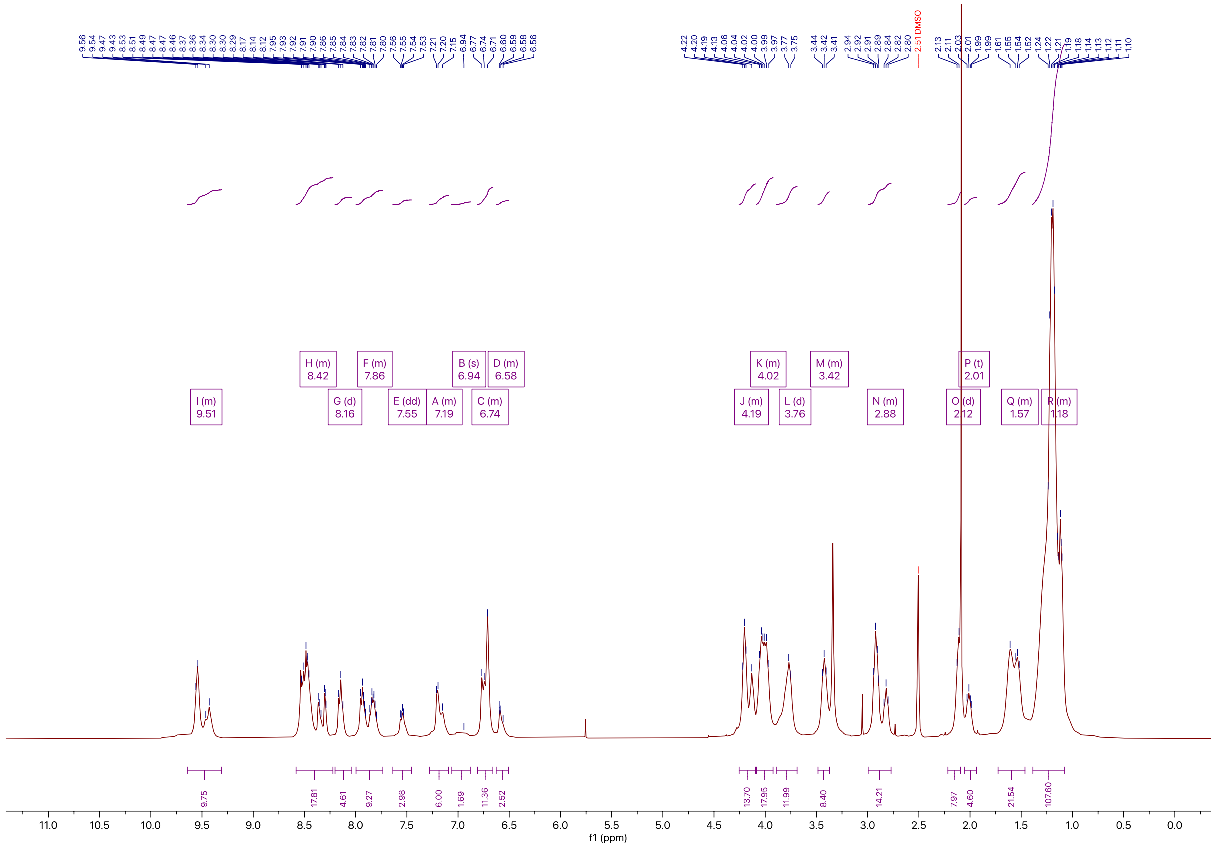

Figure S27. ^{1}H NMR spectrum (500 MHz, DMSO-d_6, 298 K) of blue fluorescent G2 Janus dendrimer **23**.

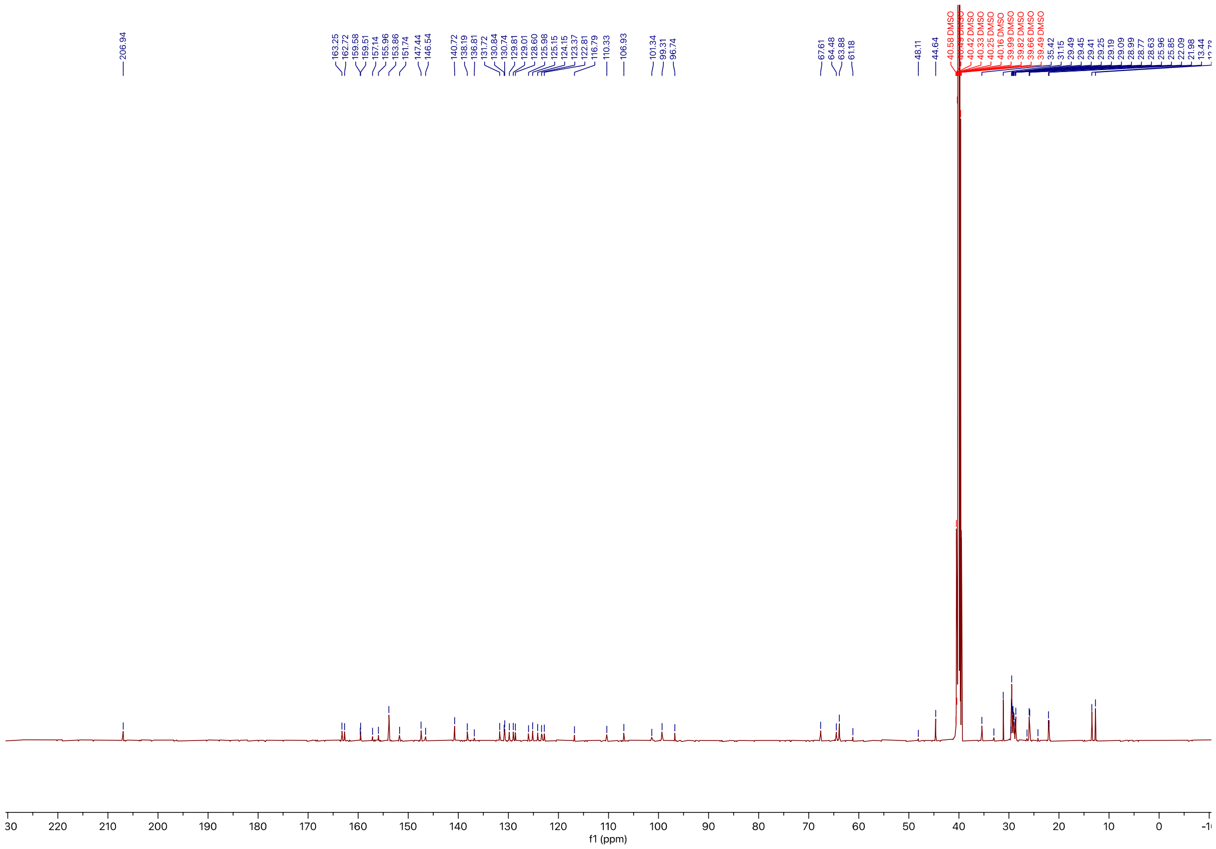

Figure S28. ^{13}C NMR spectrum (126 MHz, DMSO-d_6, 298 K) of blue fluorescent G2 Janus dendrimer **23**.

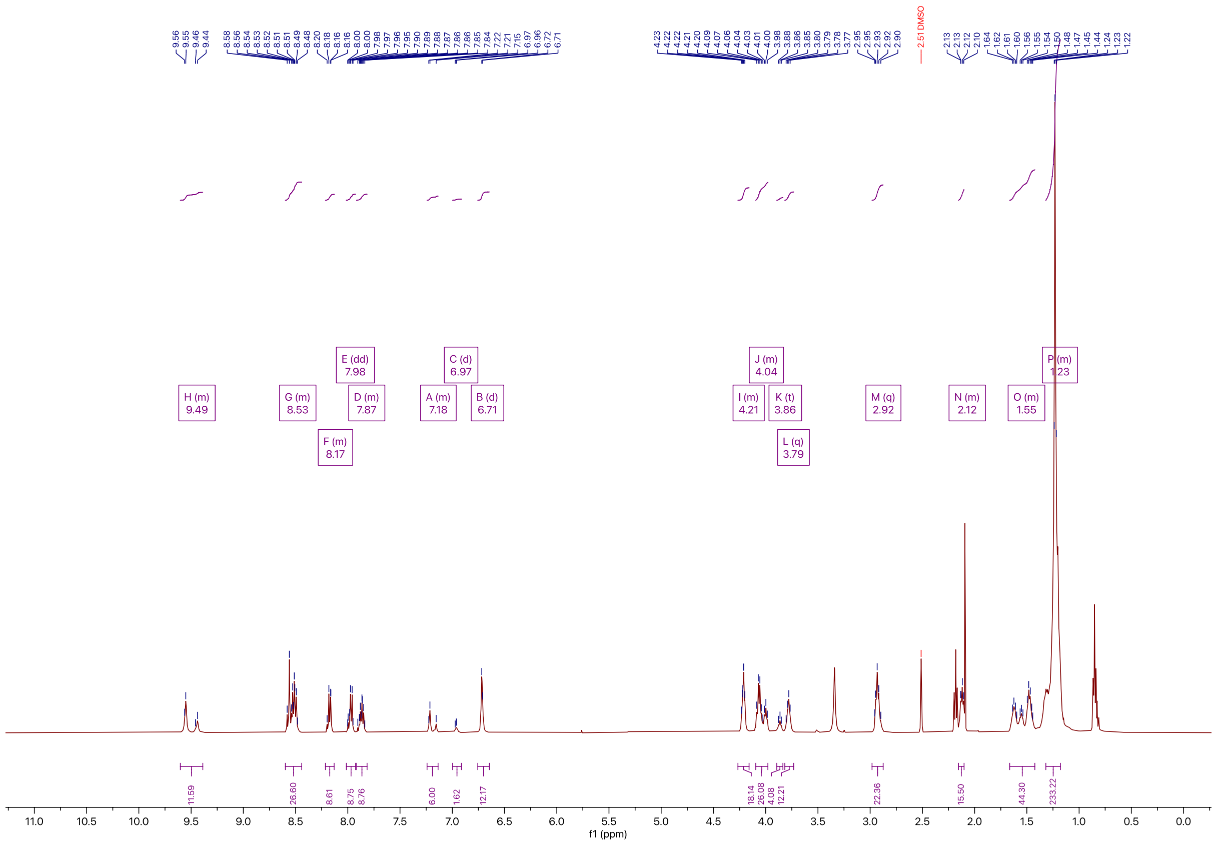

Figure S29. ^{1}H NMR spectrum (500 MHz, DMSO-d_6, 298 K) of naphthalimide-clicked G2 dendrimer **24**.

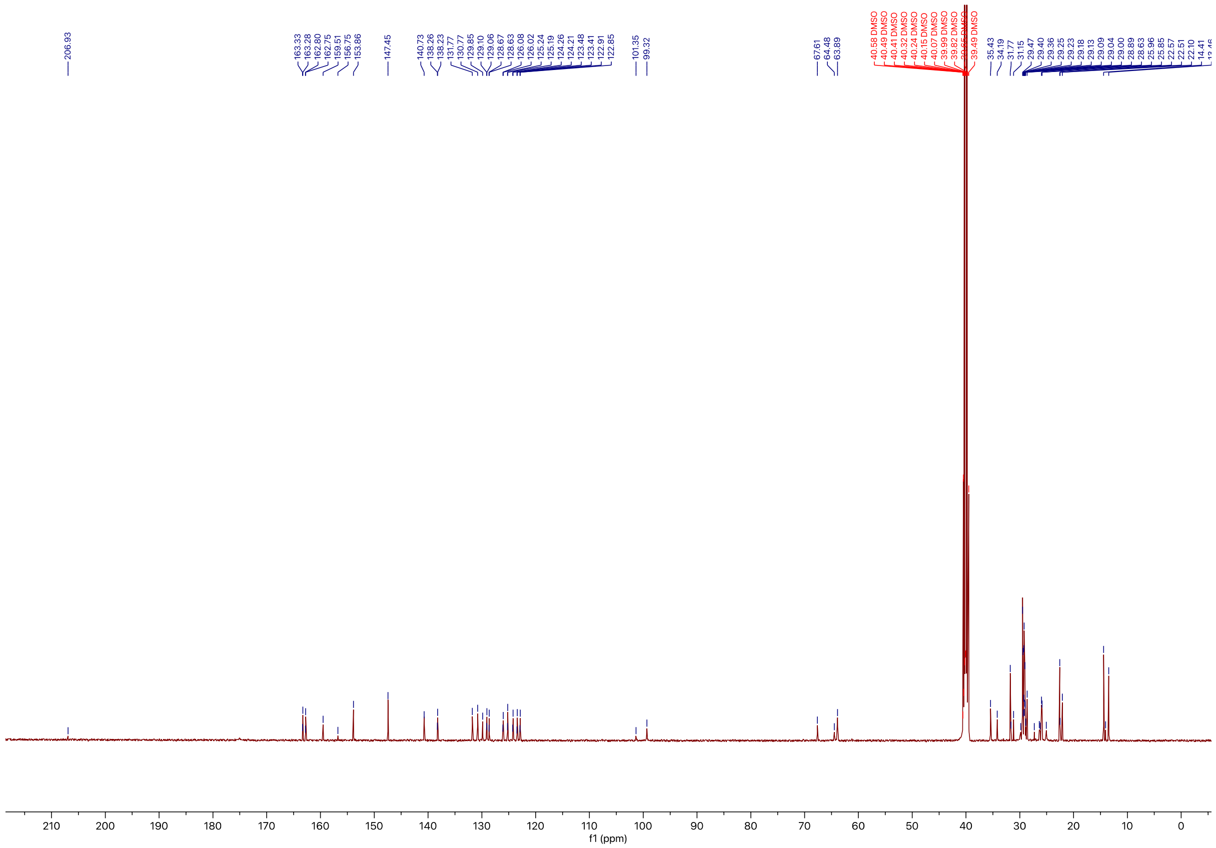

Figure S30. ^{13}C NMR spectrum (126 MHz, DMSO-d_6, 298 K) of naphthalimide-clicked G2 dendrimer **24**.

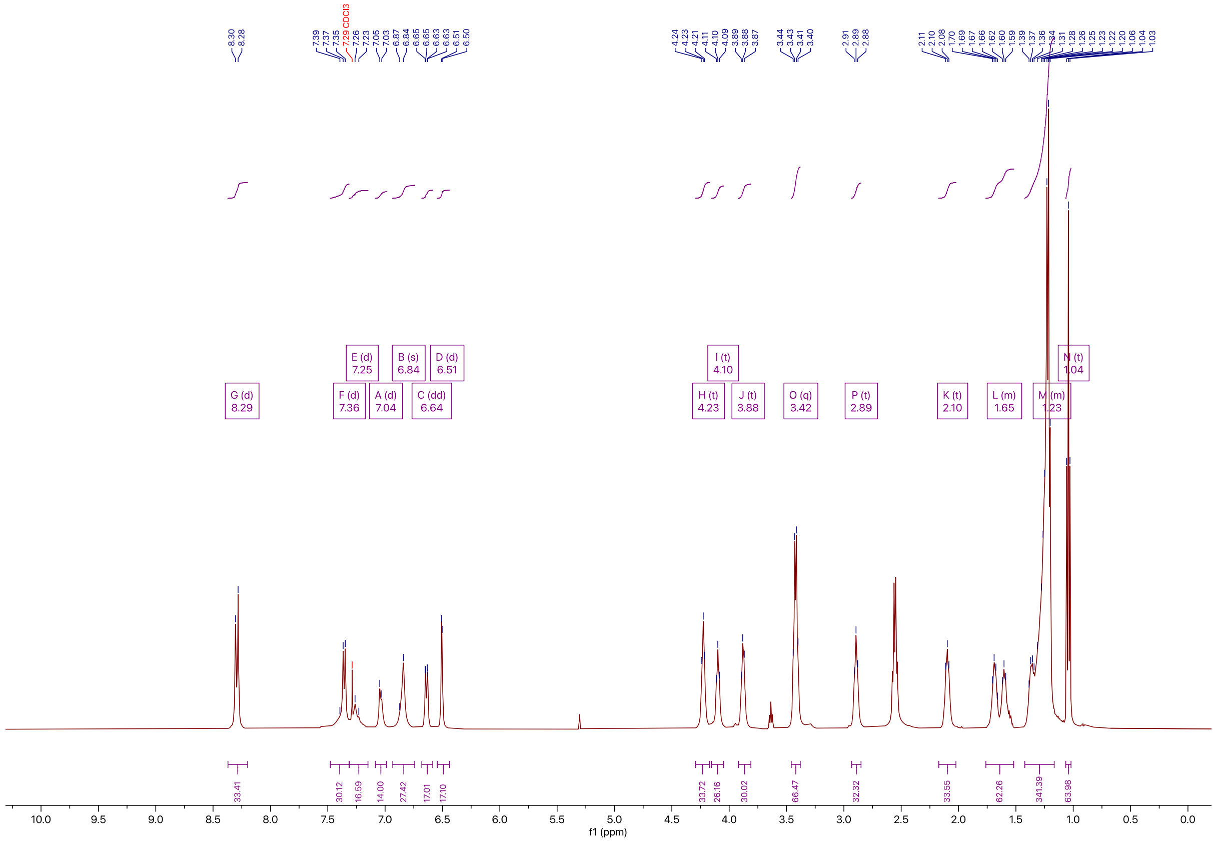

Figure S31. ^{1}H NMR spectrum (500 MHz, $CDCl_3$, 298 K) of blue fluorescent G3 dendrimer **25**.

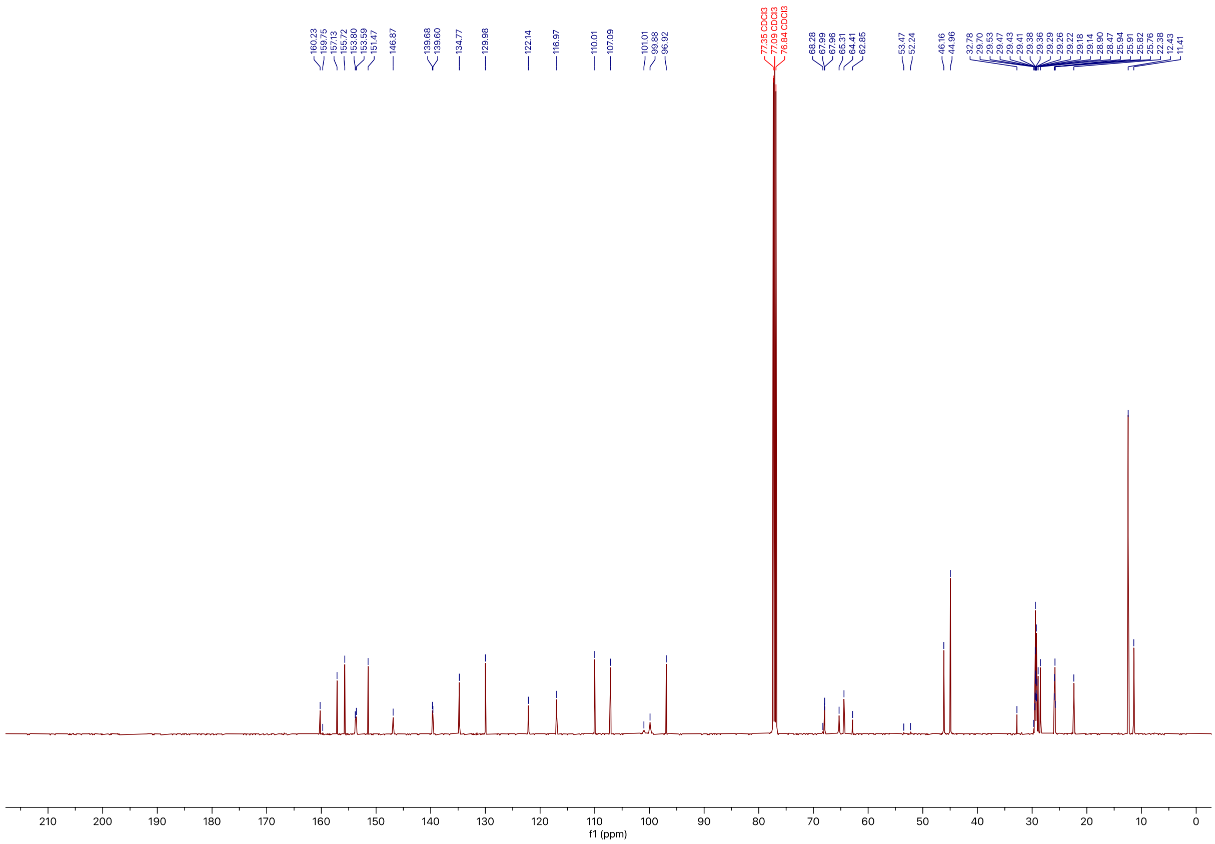

Figure S32. ^{13}C NMR spectrum (126 MHz, $CDCl_3$, 298 K) of blue fluorescent G3 dendrimer **25**.

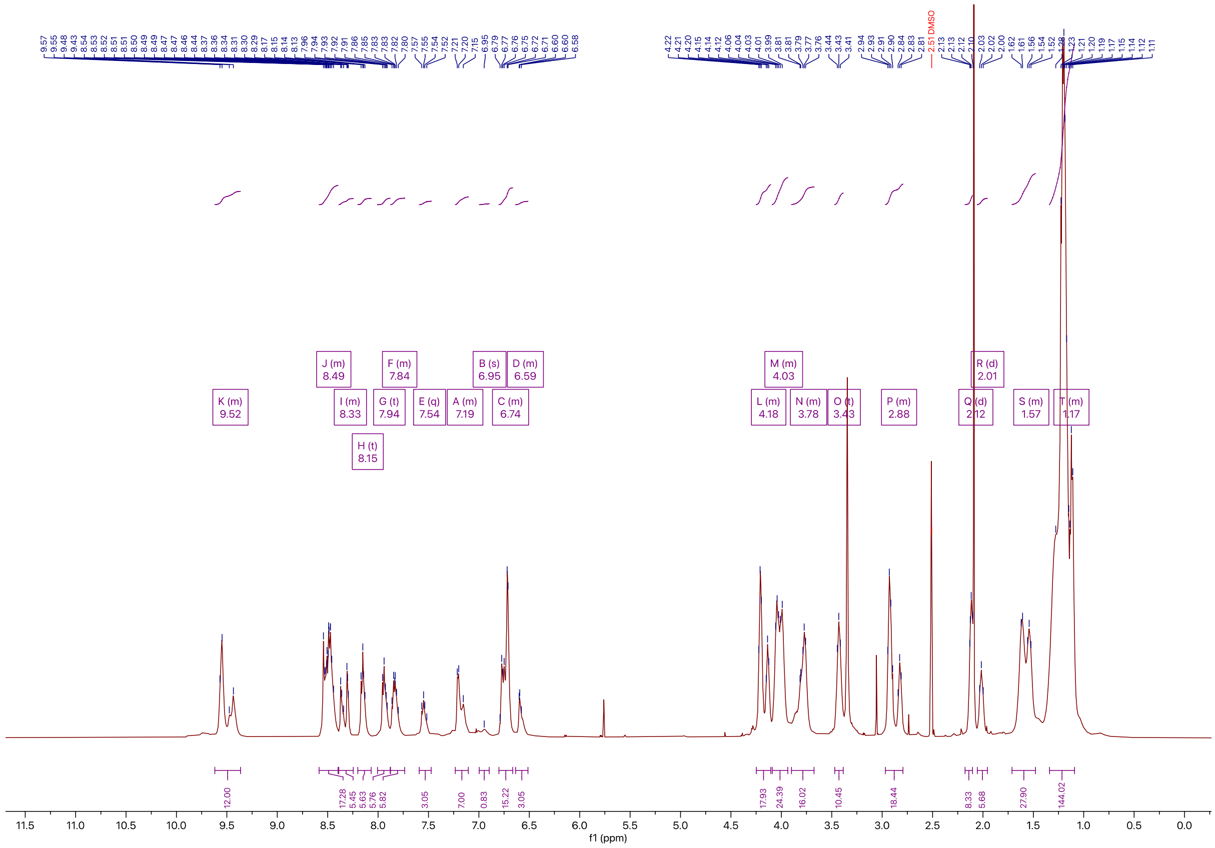

Figure S33. ^{1}H NMR spectrum (500 MHz, DMSO-d_6, 298 K) of blue fluorescent G3 Janus dendrimer **26**.

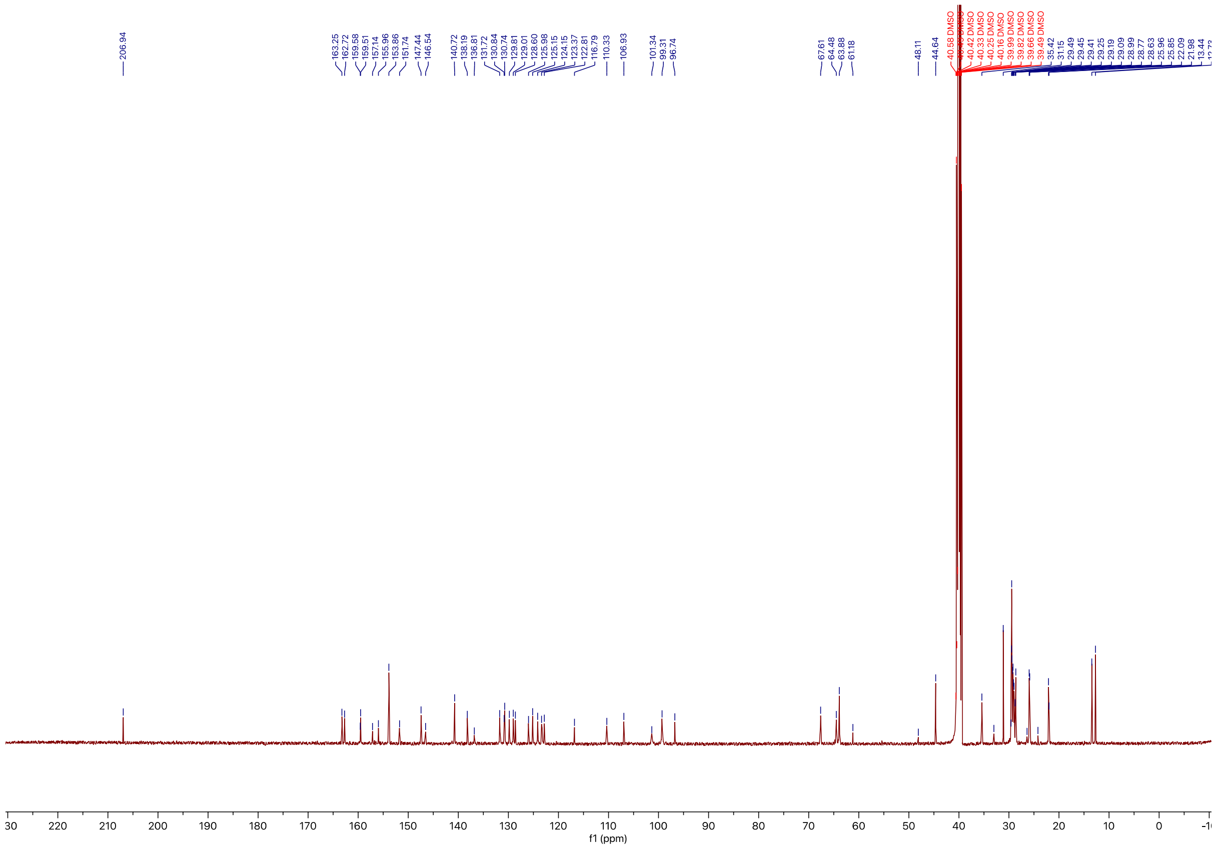

Figure S34. ^{13}C NMR spectrum (126 MHz, DMSO-d_6, 298 K) of blue fluorescent G3 Janus dendrimer **26**.

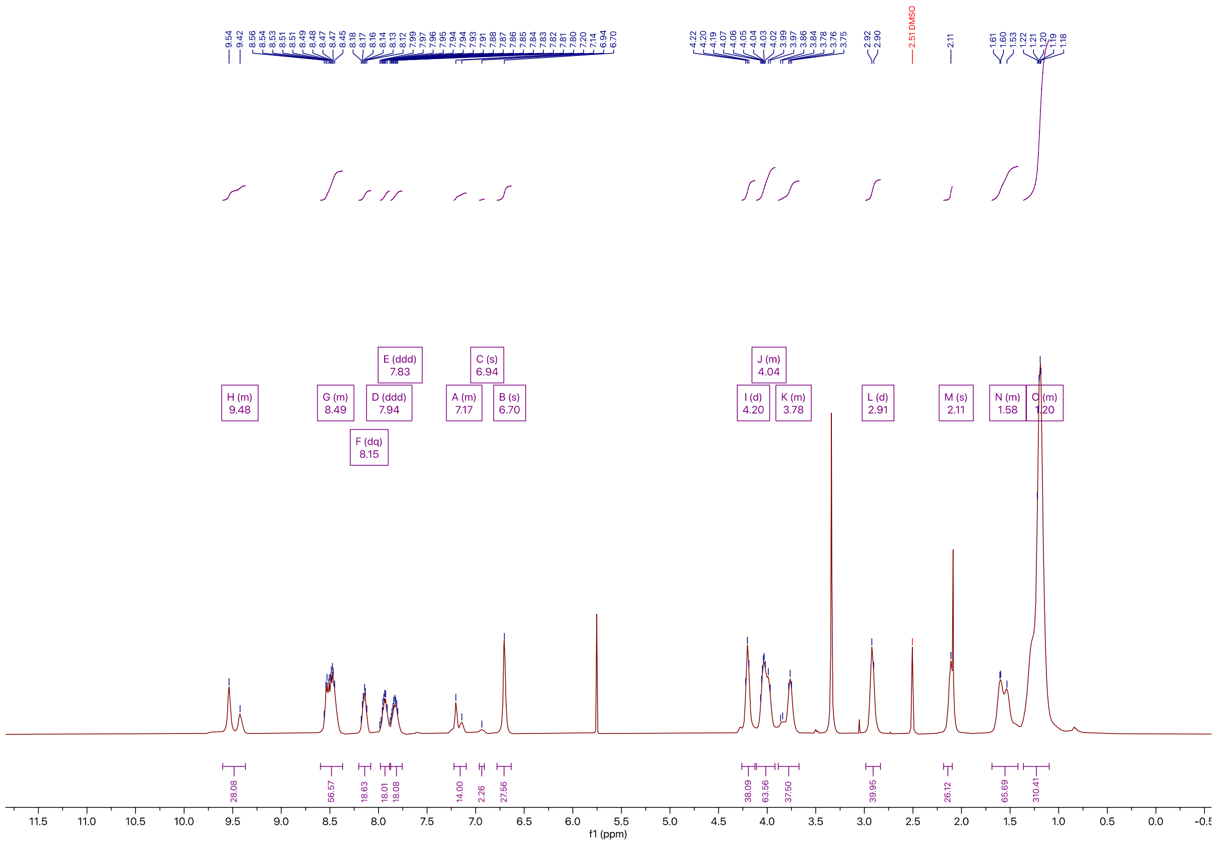

Figure S35. ^{1}H NMR spectrum (500 MHz, DMSO-d_6, 298 K) of naphthalimide-clicked G3 dendrimer **27**.

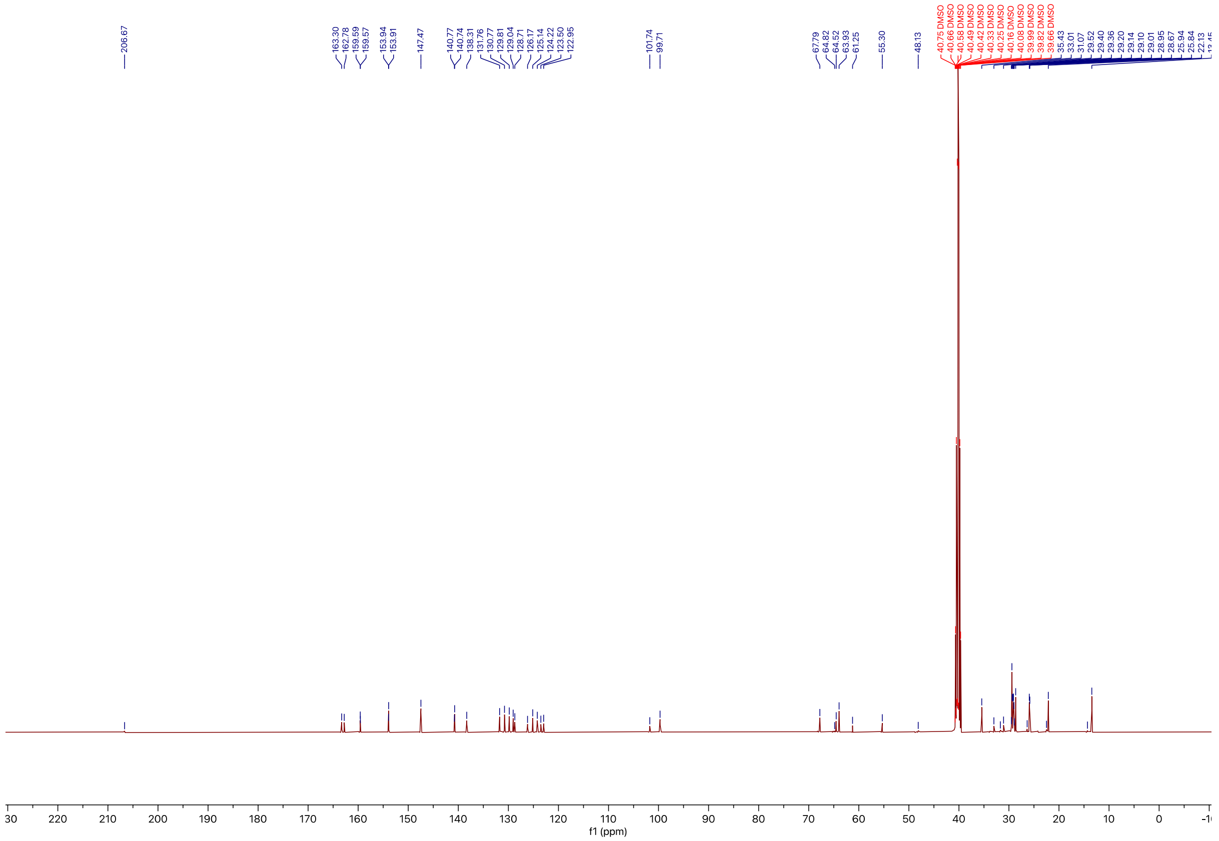

Figure S36. ^{13}C NMR spectrum (126 MHz, DMSO-d_6, 298 K) of naphthalimide-clicked G3 dendrimer **27**.
